# Pollution, stress response, and obesity: A systematic review

**DOI:** 10.1111/obr.13895

**Published:** 2025-01-17

**Authors:** Sandra El Kouche, Sarah Halvick, Chloe Morel, Radu‐Corneliu Duca, An van Nieuwenhuyse, Jonathan D. Turner, Nathalie Grova, David Meyre

**Affiliations:** ^1^ Inserm UMR 1256 Nutrition‐Genetics‐Environmental Risk Exposure (N‐G‐ERE) University of Lorraine Nancy France; ^2^ Department of Health Protection, Unit Environmental Hygiene and Human Biological Monitoring National Health Laboratory (LNS) Dudelange Luxembourg; ^3^ Department of Public Health and Primary Care, Environment and Health KU Leuven (University of Leuven) Leuven Belgium; ^4^ Department of Health Protection National Health Laboratory (LNS) Dudelange Luxembourg; ^5^ Immune Endocrine Epigenetics Research Group, Department of Infection and Immunity Luxembourg Institute of Health Esch‐sur‐Alzette Luxembourg; ^6^ Department of Health Research Methods, Evidence, and Impact McMaster University Hamilton Ontario Canada

**Keywords:** obesity, pollution, stress, systematic review

## Abstract

Limited literature addresses the association between pollution, stress, and obesity, and knowledge synthesis on the associations between these three topics has yet to be made. Two reviewers independently conducted a systematic review of MEDLINE, Embase, and Web of Science Core Collection databases to identify studies dealing with the effects of semi‐volatile organic compounds, pesticides, conservatives, and heavy metals on the psychosocial stress response and adiposity in humans, animals, and cells. The quality of papers and risk assessment were evaluated with ToxRTool, BEES‐C instrument score, SYRCLE's risk of bias tool, and CAMARADES checklist. A protocol for the systematic review was registered on PROSPERO. Of 1869 identified references, 63 were eligible after title and abstract screening, 42 after full‐text reading, and risk of bias and quality assessment. An important body of evidence shows a positive association between pollution, stress response, and obesity. Pollution stimulates the hypothalamic–pituitary–adrenal axis by activating the glucocorticoid receptor signaling and transcriptional factors responsible for adipocyte differentiation, hyperphagia, and obesity. Endocrine‐disrupting chemicals also alter the Peroxisome Proliferator‐activated Receptor gamma pathway to promote adipocyte hyperplasia and hypertrophy. However, these associations depend on sex, age, and pollutant type. Our findings evidence that pollution promotes stress, leading to obesity.

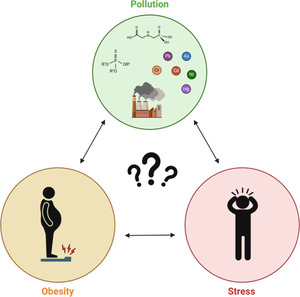

**Abbreviations**
2,3,7,8‐TCDD: 2,3,7,8‐tetrachlorodibenzo‐p‐dioxin
Adipoq: adiponectin
ADX: bilateral adrenalectomy
AhR: aryl hydrocarbon receptor
BDE‐47: 2,2,4,4‐tetrabromodiphenyl ether
BPDP: tert‐butylphenyl diphenyl phosphate
CRH: corticotropin‐releasing hormone
DEHP: bis(2‐ethylhexyl) phthalate
DINP: di‐iso‐nonyl phthalate
dw: dry weight
EHDP: 2‐ethylhexyl diphenyl phosphate
FABP: fatty acid binding protein
Fasn: fatty acid synthase
hADSC: human adipose‐derived stromal cells
IL‐6: interleukin‐6
iNos2: nitric oxide synthase 2
IRS‐1: insulin receptor substrate‐1
LPL: lipoprotein lipase
lw: lipid weight
MDA‐kb2: human breast cancer cells
MPC: mitochondrial pyruvate carrier
MTP: methamidophos
NO_2_
: nitrogen dioxide
O_3_
: ozone
p,p’‐DDT: dichlorodiphenyltrichloroethane
PAH: polycyclic aromatic hydrocarbons
PCB: polychlorinated biphenyls
PM: particulate matter
PPARγ: peroxisome proliferator‐activated receptor gamma
QpE: Quizalofop‐p‐Ethyl
SCD‐1: Stearoyl‐CoA Desaturase
si: small interfering
StAR: steroidogenic acute regulatory
TBEP: tris (2 butoxyethyl) phosphate
TOCP: tri‐o‐cresyl phosphate
WHO: World Health Organization

## 1 INTRODUCTION

The World Health Organization (WHO) defines obesity as an abnormal or excessive fat accumulation that presents a health risk. Obesity is the most prevalent non‐infectious chronic disease globally, representing a major public health issue.^1^ As an illustration, 160 million children and 890 million adults will be living with obesity in 2022.

Obesity, a non‐communicable disease, is also a risk factor for the onset of multiple co‐morbidities such as depression, sleep disorders, osteoarthritis, type 2 diabetes, non‐alcoholic fatty liver disease, hypertension, cardiovascular diseases, several types of cancer, and even viral disease (like, COVID‐19 complications).^2^ Furthermore, it is associated with stigmatization,^3^ education and employment discrimination,^3^ reduced work productivity,^4^ poverty,^5^ and social isolation.^6^ It also leads to a lower quality of life,^7^ both from physical and psychosocial perspectives. Obesity is associated with premature mortality and can lead to a 20‐year reduction in life expectancy in its most extreme forms.^8^ Obesity accounts for 5 million deaths annually^9^ and is the fifth most common leading cause of death worldwide.^10^ Despite the availability of different treatments (diet and exercise, behavioral and cognitive therapy, medication, and bariatric surgery)^11^, ^12^ and major investments in health expenditures,^13^ there is no sign of obesity prevalence slowing down worldwide.^14^ In this context, the investigation of the causes, consequences, prediction, prevention, and treatments of obesity may help in fighting the disease epidemic worldwide.^15^

Obesity stems from an interplay between biological factors (e.g., age, sex, ethnicity, pre‐existing diseases, in utero programming,^16^ gut microbiome, genetics, epigenetics) and environmental factors (e.g., diet,^17^ physical inactivity, psychosocial stress, pollution^18^).^19^ Growing evidence suggests that outdoor and indoor pollutants contribute to obesity, especially through endocrine disruptors^20^ with obesogenic action. The World Health Organization defines these contaminants as an exogenous substance or combination that alters endocrine functions. Consequently, it causes adverse health effects in an intact organism, its progeny, or (sub)populations, such as particulate matters (e.g., PM2.5, PM10), heavy metals (e.g., lead, mercury, cadmium), and persistent organic compounds (e.g., biocides, flame retardants, polycyclic aromatic hydrocarbons [PAH]).^21^ Multiple mechanisms have been proposed to explain how exposure to endocrine disruptors may alter the endocrine system and metabolism and interfere with the function of adipocytes to promote obesity.^22^ One of the mechanisms linking endocrine disruptors to obesity is the alteration of the stress response via the modulation of the glucocorticoid signaling pathway.^23^, ^24^

While multiple studies have investigated the association between pollution and stress,^25^ pollution and obesity,^26^ and stress and obesity,^27^ limited literature data have established the triangular links between pollution, stress, and obesity. To our understanding, exhaustive knowledge synthesis efforts on the links between these three topics have yet to be made. This led us to perform a systematic review of the literature with the aim to investigate the links between pollution, stress, and obesity.

## 2 METHODS

A protocol for this systematic review was registered on PROSPERO (ID: CRD42024515683 for human studies and ID: CRD42024516641 for animal studies). Criteria for search methods, article eligibility, and factors associated with pollutants, stress, and obesity traits were determined a priori. The PRISMA statement was used to guide the reporting of this systematic review (Table S1) (Huang et al 2021).

### 2.1 Study inclusion/exclusion criteria

Any study dealing with endocrine‐disrupting indoor and outdoor pollutants (e.g., semi‐volatile organic compounds, pesticides, conservatives, heavy metals), alteration of the stress response, and obesity traits were included. Human, animal, and cellular studies published in English were included. Review articles, commentaries without original data, and conference abstracts were excluded.

### 2.2 Electronic search strategy

Search strategies were developed in collaboration with the field experts (RCD, NG, JDT, DM) to systematically search the MEDLINE, Embase, and Web of Science Core Collection databases from the database's inception to February 22nd, 2024 (Table S2). Search terms such as pollutant, stress, and obesity‐related terms were used with Boolean operators to identify studies linking pollution exposure, response to stress, and susceptibility to obesity. Works cited in eligible articles were hand‐searched to ensure all pertinent studies were included in the current review.

### 2.3 Study selection

In a training exercise, the reviewers and two field experts screened a sample of 200 abstracts. Then, two reviewers independently screened article titles and abstracts to determine eligibility after removing duplicate records. Articles declared relevant by either of the reviewers were selected for full‐text review. Any disagreements at any stage of the process were resolved through consensus or consulting the field experts, as required.

### 2.4 Data extraction and management

Data were extracted in duplicate from studies meeting the inclusion criteria using a standardized form. Information on study characteristics (author's name, year, article title, DOI; study type, e.g., cross‐sectional, prospective, retrospective, case–control, case‐only, case report, pedigree, general population), model (human, animal or cell line), demographics of study participants in both comparator groups (ethnicity/geographic origin, sample size, male to female ratio, mean age with standard deviation), information on pollutant studied (name[s], concentration, exposure mode, duration), stress variable measured, obesity status and anthropometric characteristics (method of body mass index reported, estimated value of body mass index along with variability in the comparator groups, estimated effect as odds ratio, risk ratio or rate ratio [if reported]), and additional clinical and biological features were noted (using the P‐value approach).

### 2.5 Risk of bias, quality assessment, and evidence levels

The quality and risk of bias of the included studies were assessed independently by two reviewers. All conflicts were resolved by discussion with the field experts. The quality of the included studies was assessed using the BEES‐C instrument for human studies and the Toxicological data reliability assessment tool (ToxRTool) for in vivo (animal and human) and in vitro studies.^28^, ^29^ The BEES‐C instrument score classified articles as follows: TIER 1 (most relevant), TIER 2 (relevant), and TIER 3 (not so relevant).^29^ The ToxRTool had a maximum score of 21 for in vivo and 18 for in vitro studies. In line with previous studies, in vivo papers were categorized on reliability as follows: useful (18‐21), potentially useful (13‐17), and not useful (<13 or not all red criteria met). In vitro articles were classified as follows: useful (15‐18), potentially useful (11‐14), and not useful (<11 or not all red criteria met).^28^, ^29^, ^30^ SYRCLE's risk of bias tool and CAMARADES checklist were used for animal studies.^31^, ^32^ SYRCLE and CAMARADES risk of bias tools follow the GRADE approach.^31^, ^32^

## 3 RESULTS

### 3.1 Literature search

**FIGURE 1:**
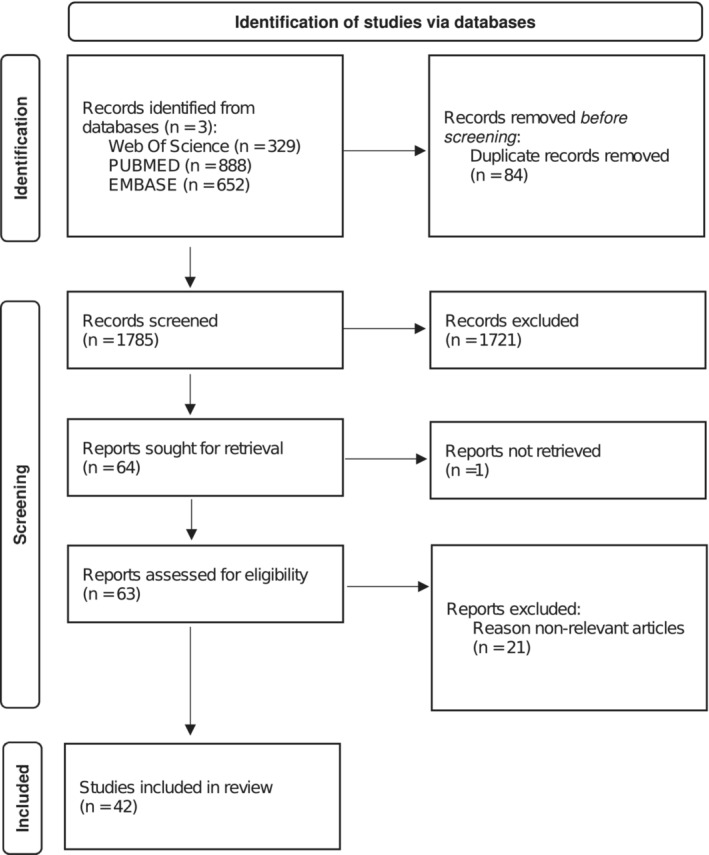
Flow chart of the selection process.

Our systematic search in Web of Science, PUBMED, and EMBASE resulted in 1869 relevant references. Then, 1785 titles and abstracts were double‐blind screened for title and abstract to determine eligibility after removing 84 duplicates. In total, 63 references were selected for full‐text review, of which 21 were deemed non‐relevant after full‐reading then excluded. Forty‐two articles were considered eligible for the literature review (Figure 1). Six, twenty‐five, and thirteen studies included human, animal, and cellular models, respectively, and 4.8% (2/42) of the eligible articles included more than one model. A total of 8500 and 3163 participants were selected in human and animal studies, respectively (Table S3).

### 3.2 Quality assessment of included studies

Four quality assessment tools were used to evaluate the 42 eligible articles. These tools consist of a series of questions or a checklist that gives each article a reliability score. All articles were assessed through ToxRTool and all in vivo and in vitro studies were reliable without restriction except for 3 articles that were only potentially useful. Human studies were also assessed through BEES‐C instrument score, and all but one article turned out to be the most relevant (TIER1). The remaining article was assessed as relevant (TIER 2). Animal studies were also evaluated through the CAMARADES checklist and SYRCLE‐tool, the former revealed 19 out of 25 studies to score above average and the latter found 5 out of 25 to score above average (Table S4).

### 3.3 Semi‐volatile organic compounds

#### 3.3.1 Ambient air pollution

##### Human studies

We included only two studies regarding the impact of ambient air pollution on stress and obesity. The first one was conducted in Los Angeles by Toledo‐Corral et al and assessed the effect of ambient air pollutants on the hypothalamic–pituitary–adrenal axis and the risk of obesity. This study was performed on 203 Latino children with overweight and obesity (8–13 years old), family history of type 2 diabetes, and had low socio‐economic status. The participants were subjected to monthly exposure to the following air pollutants: ozone (O_3_) (37.4 ppm), particulate matter (PM) (23 ppm), and nitrogen dioxide (NO_2_) (32.8 ppm, for a duration of 12 months, and fasting morning serum cortisol concentration was analyzed. Regardless of sex and pubertal status, the results showed high circulating serum cortisol levels associated with higher short‐term (1–7 months) chronic exposure to O_3_ (β = 0.06, 95% CI:0.003, 0.12, p = 0.04), while the opposite was revealed with higher long‐term (4–10 months) exposure to particulate matter and NO_2_ (β = −0.26, 95% CI:‐0.48, −0.04, p = 0.02 and β = −0.14, 95% CI:‐0.27, −0.01, p = 0.04, respectively). The data demonstrate that hypothalamic–pituitary–adrenal axis is affected by ambient air pollution^33^ (Table 1).

**TABLE 1:** In vivo studies recounting the effects of semi‐volatile organic compounds.

Type of SVOC	Pollutants and concentrations	Species/ethnicity/age	Results	References
Ambient air pollution	Particulate matter (PM_2.5_): 23 μg/m^3^ exposition for 12 months	Human: Overweight and obese Latino children (8–13 years old)	Chronic exposure to PM induces HPA‐axis dysregulation PM causes low morning serum cortisol PM's effect is independent of sex and pubertal status	Toledo‐Corral et al, 2021
Ambient air pollution	Detection of 30.2 μg/m^3^ PM_2.5_ and 26.6 μg/m^3^ PM_10_ during the first trimester	Human: Chinese pregnant women (28.6 years old)	PM exposure during the first trimester of pregnancy reduces fetal growth by increasing fetal cortisol	Li et al, 2023
HAH	TCDD: 50 μg/kg body weight, single IP injection	Animal: 6–8‐year‐old rats (Wistar)	TCDD induces hypoglycemia TCDD inhibits the activity of adrenal corticosteroid‐synthesizing enzymes	Jovanovich et al, 1987
HAH	TCDD: 0.036 μg/kg (on dose, orally)	Animal: 60 days old pregnant mice (C57Bl/6 J)	TCDD reduces adiponectin When mixed with PFOA, BPA, and atrazine, treatment for 12 days induced maternal glucose tolerance alteration	Merrill et al, 2021
Flame retardants	PCB: Detection of 48.7 to 71.5 ng/g lw	Human: Obese Norwegian patients (18–60 years old)	Increased blood level of PCB after bariatric surgery PCB increase is associated with serum cortisol reduction PCB affects HPT‐ and HPG‐axes PCB affects testosterone in males	Jansen et al, 2020
Flame retardants	BPA: Detection of 0.81 ng/ml in urinary	Human: Chinese participants, lean (61.9 ± 9.3 years) and obese (63.4 ± 9.5 years)	BPA is associated with inflammation in lean subjects BPA induces higher expression of inflammatory cytokines in lean women Obesity attenuates BPA‐mediated‐chronic inflammation	Yang et al, 2016
Flame retardants	Mirex: 40 or 20,000 mg/l, IP for 4 days	Animal: 6–8‐year‐old rats (Wistar)	Mirex induces hypoglycemia and hyperlipidemia Mirex inhibits the activity of adrenal corticosteroid‐synthesizing enzymes Mirex does not affect β‐cells	Jovanovich et al, 1987
Flame retardants	Perfluorooctane sulfonic acid (PFOS): 1 and 5 mg/kg bw/day for 17 days	Animal: Pregnant 12 weeks old rats (Sprague–Dawley)	PFOS reduces fetal weight in a dose‐dependent manner PFOS alters hormone levels and increases corticosterone PFOS alters steroidogenic enzymes in a sex‐dependent manner	Dangudubiyyam et al, 2023
Flame retardants	BPA: 100 μg/kg/day IP for 13 days	Animal: Pregnant rats (Sprague–Dawley)	BPA increases body and fat weight, insulin resistance, hyperlipidemia, hepatic lipid deposition, and fasting blood glucose in male offspring BPA‐induced GR‐ and PPARγ‐pathways alteration in exposed rats	Huang et al, 2021
Flame retardants	BPA: 5 to 5000 μg/kg/day for 30 days	Animal: Adolescent mice (5‐week‐old, C57Bl/6 J)	BPA increases inflammation, body weight, and fat mass in low‐calorie‐diet‐fed mice BPA promotes adipogenesis through partial activation of GR	Yang et al, 2016
Flame retardants	BPA: 50 μg/kg/per os for 3 weeks	Animal: Obese adult male mice (6 weeks old, C57Bl/6 J)	BPA worsens the effects of high‐fat diet BPA induces obesity, anxiety, neuroinflammation, and hyperactivation of the HPA‐axis	Lama et al, 2023
Flame retardants	TCDD: 20 pg/kg/bw/day; PCB: 200 ng/kg/bw/day; DEHP: 500 μg/kg/bw/day; BPA: 40 μg/kg/bw/day	Animal: Mice (4‐week‐old, C57Bl/6 J)	Low dose mixture of pollutants induce metabolic changes in mice Pollutants affect GR signaling in VAT and letpin synthesis in female Pollutants affect GR signaling in SAT male GR signaling not affected in liver Circadian clock may modulate the pollutants' effects	Vega et al, 2021
Flame retardants	BPA: 50 μg/kg; perfluorooctanoic acid (PFOA): 0.1 mg/kg (1 dose, orally)	Animal: 60 days‐old pregnant mice (C57Bl/6 J)	PFOA reduces adiponectin Chemicals alone did not affect glucose tolerance, body weight, and corticosterone levels When mixed with TCDD and atrazine, treatment for 12 days induced maternal glucose tolerance alteration	Merrill et al, 2021
Flame retardants	TBBPA, BDE, and BPS: 0,2 mg/kg/bw/day, from pregnancy day 8 through postpartum day 21	Animal: Pregnant mice (CD‐1)	Pollutants affect social behavior: increased anxiety and reduced social contacts BDE and BPS decreased body weight in males Higher body weight is associated with lower social hierarchy rank Body weight is a causative factor for social ranking	Kim et al, 2015
Flame retardants	PCB: 1 dose of 0.5 mg/kg bw; BDE: 7 mg/kg bw/week for 1 year	Animal: *Felis catus* (Japan) males (24–28 months old for PCB and 13 months old for BDE)	PCB did not affect body weight PCB increased corticosterone levels and testis weight Male reproductive system may be a target for PCB BDE increased weight, fat, triglycerides, and HDL	Khidkhan et al, 2023
Flame retardants	PCB 153: 98 μg/kg/bw/day; PCB 118: 49 μg/kg/bw/day	Animal: Pregnant Norwegian white ewes (2–7 years old)	PCB affects the development of the adrenal cortex and fetal cortisol PCB increased plasma ACTH in female fetuses PCB‐induced lower plasma cortisol, body weight, and hypotrophic adrenal cortices in exposed fetuses	Zimmer et al, 2013

ACTH: Adreno corticotropic hormone; BDE: 2,2,4,4‐tetrabromodiphenyl ether; BPA: Bisphenol A; BPS: Bisphenol S; DEHP: di‐[2‐ethylhexyl]‐phthalate; GR: Glucocorticoid receptor; HAH: Halogenated aromatic hydrocarbons; HDL: High density lipoprotein; HPA: hypothalamic–pituitary–adrenal; HPG: hypothalamic–pituitary–gonadal; HPT: hypothalamic–pituitary‐thyroid; IP: intraperitoneal; lw: lipid weight; PBDE: polybrominated biphenyl esters; PCB: polychlorinated biphenyls; PM: particulate matter; PFOA: perfluorooctanoic acid; PFOS: Perfluorooctane sulfonic acid; PPARγ: Peroxisome proliferator‐activated receptor γ; SAT: subcutaneous adipose tissue; TBBPA: tetrabromobisphenol‐A; TCDD: 2,3,7,8‐Tetrachlorodibenzo‐p‐dioxin; VAT: visceral adipose tissue.

The second study investigated the association between particle matter exposure, cortisol levels, and birth outcomes in a prospective cohort of 7419 pairs of 28.6‐year‐old pregnant Chinese women with a body mass index of 21.5 kg/m^2^ and their offspring. The participants were exposed to 30.2 μg/m^3^ of PM_2.5_ and 26.6 μg/m^3^ of PM_10_ during the first trimester of pregnancy. Results revealed a negative association between the abdominal circumference's z‐score and particle matter exposure, especially in the third trimester (β = −0.107, 95% CI:‐0.154, p = 0.06 and β = −0.089, 95% CI:‐0.135, p = 0.043 for PM_2.5_ and PM_10_, respectively). Additionally, birth weight's z‐score was negatively associated with particulate matter exposure during the first and second trimesters. In the first trimester, birth weight's z‐score was reduced by β = −0.54, 95% CI:‐0.09, p = 0.017 and β = −0.038, 95% CI:‐0.072, p = 0.003, for PM_2.5_ and PM_10_, respectively. In the second trimester, it was reduced by β = −0.053, 95% CI:‐0.087, p = 0.02 and β = −0.047, 95% CI:‐0.078, p = 0.015 for PM_2.5_ and PM_10_, respectively. Cord and maternal cortisol were measured, and the results indicated a positive association of cord blood cortisol in the first trimester with PM_2.5_ and PM_10_ exposure by a z‐score of β = 0.082, 95% CI:‐0.029, p = 0.135, and β = 0.086, 95% CI:‐0.036, p = 0.136, respectively. At the same time, maternal cortisol before delivery was negatively associated with PM_2.5_ and PM_10_ exposure by a z‐score of β = −0.0019, 95% CI:‐0.049, p = 0.012, and β = −0.0018, 95% CI:‐0.047, p = 0.011, respectively. Interestingly, cord blood cortisol was negatively associated with the birth weight's z‐score (β = −0.17, 95% CI:‐0.23, p = 0.10). In the first trimester, ambient air pollution exerted indirect effects on neonatal parameters (birth weight, length, and head circumference) through cord blood cortisol mediation by 20.62%, while in the first‐second trimester, the proportion of mediation by cortisol is 13.90%. Hence, these data suggest that air pollution strongly affects the hypothalamic–pituitary–adrenal axis and fetal growth, especially during the early exposition^34^ (Table 1).

#### 3.3.2 Halogenated aromatic hydrocarbons

##### Animal studies

Two animal studies and one cellular study on halogenated aromatic hydrocarbons complied with our study criteria. Jovanovich et al investigated hypoglycemia and hyperlipidemia phenotypes in 6–8‐week‐old rats injected intraperitoneally with 2,3,7,8‐tetrachlorodibenzo‐p‐dioxin (2,3,7,8‐TCDD) (50 μg/kg body weight). In female rats, 2,3,7,8‐TCDD was associated with slight weight loss (1.01‐fold), reduced food consumption (1.2‐fold), decreased serum glucose (1.5‐fold), increased serum triglycerides (2.25‐fold), and a decreased adrenocorticoid activity (1.8‐fold for Δ‐4‐isomerase and 1.35‐fold for 11‐β‐hydroxylase). The authors concluded that alteration of the glucocorticoid pathway caused by halogenated aromatic hydrocarbons such as 2,3,7,8‐TCDD promotes lipid storage and glycogen synthesis^35^ (Table 1).

The effects of 2,3,7,8‐TCDD on maternal metabolic health were studied in 60‐day‐old pregnant C57Bl/6 mice that were either fed a single dose of 0.036 μg/kg of 2,3,7,8‐TCDD or a mixture containing 0.036 μg/kg of 2,3,7,8‐TCDD, 10 mg/kg of atrazine, 0.1 mg/kg of perfluorooctanoic acid, and 50 μg/kg of bisphenol A (BPA) for 12 days. 2,3,7,8‐TCDD alone had no effect on body weight, corticosterone levels, triglycerides, high‐density lipoprotein and low‐density lipoprotein levels, glucose tolerance, cholesterol levels, body volume, visceral adipose volume, resistin, MCP‐1, insulin, and PAI‐1 levels. In contrast, 2,3,7,8‐TCDD exposure increased adiponectin level with a Cohen's d of 2.67. Furthermore, when 2,3,7,8‐TCDD is mixed with bisphenol A, perfluorooctanoic acid, and atrazine, results were similar to 2,3,7,8‐TCDD treatment alone, except for an increased low‐density lipoprotein level and hyperglycemia with a Cohen'd of 1.73 and 2.75, respectively. Interestingly, the mixture of chemicals did not affect adiponectin levels in pregnant and non‐pregnant mice. The authors, therefore, confirm that endocrine‐disrupting chemicals result in the alteration of maternal glucose, especially during pregnancy^36^ (Table 1).

A recent study exposed mice to a mixture of pollutants (20 pg/kg/bw/day of 2,3,7,8‐TCDD, 200 ng/kg/bw/day of polychlorinated biphenyls (PCB), 500 μg/kg/bw/day of bis(2‐ethylhexyl) phthalate (DEHP) and 40 μg/kg/bw/day of bisphenol A) to assess the link between pollutants and glucocorticoid signaling in mice. The authors demonstrated a decreased mRNA expression of the glucocorticoid receptor (1.4‐fold), leptin plasma levels (2‐fold), and canonical genes involved in the circadian clock machinery in the visceral adipose tissue of female mice only. In summary, a strong effect of a mixture of pollutants, including 2,3,7,8‐TCDD, was observed on glucocorticoid signaling. This study also suggests that the effect is sex‐ and tissue‐dependent and alters the clock machinery^37^ (Table 1).

##### Cellular and molecular study

The effects of 2,3,7,8‐TCDD were also studied on adipose differentiation. In 1995, Phillips et al demonstrated that 2,3,7,8‐TCDD treatment (3 μg) on preadipose 3 T3 clonal cells before or during the first two days of differentiation resulted in a significant reduction in adipose cell colonies (representing approximately 20% of control), adipocyte‐specific mRNAs such as lipoprotein lipase (LPL), adipocyte fatty acid binding protein (FABP, also known as aP2), and adipsin. The ability of 2,3,7,8‐TCDD to bind to the Ah receptor was tested with an Ah receptor antagonist, the results highlighting a significant competition for the binding site (partial block of 2,3,7,8‐TCDD induced by a 1000‐fold molar excess of αNF). These results demonstrate that the action of 2,3,7,8‐TCDD is mediated by the Ah receptor and interferes with the signaling pathways of inducing agents (dexamethasone and isobutylmethylxanthine), thereby suggesting a possible direct effect on glucocorticoid‐induced differentiation factors. Moreover, the activity of protein kinase A was increased two times in the presence of 2,3,7,8‐TCDD, suggesting that 2,3,7,8‐TCDD may act as a disruptor of the cAMP pathway^38^ (Table 2).

**TABLE 2:** In vitro studies recounting the effects of semi‐volatile organic compounds.

Type of SVOC	Pollutants and concentration	Cell type	Results	References
HAHs	TCDD: 10 nM for 3 days	3 T3‐L1 adipocytes	Cells treated with dexamethasone, isobutylmethylxanthine, and TCDD before or in the first two days of differentiation induces a number of fat cell colonies reduction TCDD's effect seems to be mediated by the AhR TCDD enhances PKA activity	Philips et al, 1995
Flame retardants	BPA: 25 or 50 μM for 12 days	Primary human preadipocytes	BPA promotes adipocyte differentiation in the absence of glucocorticoid BPA is mediated through a non‐classical ER pathway rather than the GR pathway BPA acts as an obesogenic	Boucher et al, 2014
Flame retardants	BPA: 10 nM, 1 and 80 μM for 24 h	Children adipocytes (3–13 years old)	BPA promotes adipogenesis and stimulates obesity during childhood through regulation of the 11β‐HSD1 enzyme 11β‐HSD1 regulation mediated by BPA involves the GR pathway	Wang et al, 2013
Flame retardants	BPA: 100 nM/l and 1 μmol/l for 3 days	3 T3‐L1 adipocytes	BPA stimulates GR activity BPA stimulates adipogenesis through the GR pathway	Sargis et al, 2010
Flame retardants	BPA: 1 and 10 nM for 6 days	3 T3‐L1 adipocytes	BPA promotes adipocyte differentiation in the absence of glucocorticoid BPA does not activate the transcriptional activity of GR BPA acts through the activation of a pathway different from the GR pathway BPA is unlikely to act as a GR or PPARγ agonist	Atlas et al, 2014
Flame retardants	TBBPA: 1.10^−5^ M to 1.10^−12^ M for 4 days	3 T3‐L1 adipocytes	TBBPA promotes adipogenesis TBBPA is a weak activator of PPARγ TBBPA affects preadipocytes before or in the first two days of differentiation TBBPA's effects are mediated by multiple pathways that may include the GR and PPARγ pathways	Chapell et al, 2018
Flame retardants	TBBPA: 10 to 20 μM for 48 h	3 T3‐L1 adipocytes	TBBPA promotes adipocyte differentiation TBBPA's effects are mediated through multiple pathways that include the partial activation of PPARγ and GR	Liu et al, 2020
Flame retardants	BBP, BPDP, DBP, DEHP, DINP, DPP, EHDP, TBEP, TOCP, TPP detected in plastic (concentrations not given)	3 T3‐L1 adipocytes	Chemicals promote adipocyte differentiation PPARγ signaling pathway is not necessarily used by all chemicals GR signaling is not used by the chemicals	Volker et al, 2022

Abbreviations: 11β‐HSD1, 11β‐Hydroxysteroid Dehydrogenase 1; AhR, Aryl hydrocarbon receptor; BPA, Bisphenol A; BBP, benzyl butyl phthalate; BPDP, tert‐butylphenyl diphenyl phosphate; DBP, dibutyl phthalate; DEHP, bis(2‐ethylhexyl) phthalate; DINP, iso‐nonyl phthalate; DPP, diphenyl phosphate; EHDP, 2‐ethylhexyl diphenyl phosphate; ER, Estrogen receptor; GR, Glucocorticoid receptor; HAH, Halogenated aromatic hydrocarbons; PKA, Protein kinase A; PPARγ, Peroxisome proliferator‐activated receptor γ; TBBPA, Tetrabromobisphenol A; TBEP, tris(2 butoxyethyl) phosphate; TCDD, 2,3,7,8‐Tetrachlorodibenzo‐p‐dioxin; TOCP, tri‐o‐cresyl phosphate; TPP, triphenyl phosphate.

#### 3.3.3 Flame retardants

##### Human studies

A prospective cohort study was recently conducted on Norwegian patients with obesity one year after they underwent bariatric surgery to investigate the association between thyroidal, reproductive, and glucocorticoid hormone concentrations, persistent organic pollutants, and weight. The results showed a positive association of lipophilic persistent organic pollutants, such polychlorinated biphenyls (48.7 ng/g lipid weight [lw] and 71.5 ng/g lw before and after surgery, respectively), and testosterone in males (51%), and a negative association with thyroid hormones (ratio rT3/fT4 of −15.5%) suggesting an effect on the hypothalamic–pituitary‐gonadal‐ and the hypothalamic–pituitary‐thyroid‐axes^39^ (Table 1).

##### Animal studies

Jovanovich et al also investigated the effects of the non‐biodegradable organochlorine insecticide mirex on rats fed either low (40 ppm) or toxic (20,000 ppm) concentration levels. Regardless of the sex, higher liver weight (1.4‐fold), lower serum glucose (1.06‐fold for males and 1.25‐fold for females), and lower activity of adrenocortical enzymes (1.25‐fold for Δ‐4‐isomerase, and 1.13‐fold for males and 1.26‐fold for females for 11‐β‐hydroxylase) were observed in low‐dose mirex‐fed rats. Increased serum cholesterol (60% in males and 62% in females) and triglyceride levels (30%) were also observed in these animals. However, the toxic concentration of mirex in female rats was associated with weight loss (1.16‐fold), reduced food consumption (2.96‐fold) and serum glucose (1.46‐fold), increased serum triglycerides (1.18‐fold), and death. These results are similar to those observed in rats injected with 2,3,7,8‐TCDD. In summary, this study demonstrates that exposure to the flame retardant mirex results in energy homeostasis disruption, hyperlipidemia, and hypoglycemia, possibly by altering the glucocorticoid pathway^35^ (Table 1).

Several studies have demonstrated a strong association between bisphenol A and obesity. Huang et al conducted a study on rat offspring birthed by pregnant rats exposed to daily doses of bisphenol A (100 μg/kg/day intraperitoneally). Offspring had a significantly higher body weight, especially the male offspring (1.2‐fold), which also exhibited hyperglycemia, hyperlipidemia, insulin resistance, and elevated circulating glucagon and free fatty acids. Moreover, the adipogenesis‐related proteins *C/EBPα*, *PPARγ*, *SREBP1c*, *LPL*, *ACC*, and *SCD‐1* mRNA levels were increased (9‐, 50‐, 100‐, 50‐, 11‐, and 10‐fold, respectively), as well as adipocyte differentiation‐associated glucocorticoid receptor protein phosphorylation (2‐fold) and inflammation‐related molecule (*CD68*) mRNA level (1.6‐fold). In contrast, the mRNA levels of the proteins *p‐HSL (Ser660)/HSL*, *PGC‐1α*, *ATGL*, and *UCP‐1*, associated with lipolysis, were decreased (1.47‐, 2‐, 1.1‐, and 3.89‐fold, respectively). Thus, this study shows an obesogenic effect of bisphenol A mediated by its actions on estrogen, glucocorticoid receptors, thyroid hormones, and peroxisome proliferator‐activated receptor gamma (PPARγ)^40^ (Table 1).

Similar findings were observed in another study in which mice were subjected to several dosages of bisphenol A (from 5 to 5000 μg/kg/d) by oral intake for 30 days. Regardless of the bisphenol A dosage and sex status, low‐calorie‐diet‐fed mice gained more weight (1.1‐fold) and fat mass (3‐fold), and had increased circulating inflammatory factors such as leptin, resistin, interleukin‐6 (IL‐6), Tnfα, IL‐1β, Ifnγ, and nitric oxide synthase 2 (iNos2). For all concentrations of bisphenol A, the level of the leptin and resistin molecules were increased by a factor of 1500 and 2, respectively, while only 5000 μg/kg of bisphenol A‐induced increased levels of IL‐6, Tnfα, IL‐1β, Ifnγ, and iNos2 by 7‐, 3‐, 10‐, 3.5‐, and 6‐fold, respectively. Age, exposure window, and genetic background were shown to influence the extent of the bisphenol A's effect. Molecular data indicate that bisphenol A induces adipocyte differentiation by activating the transcriptional cascade of the adipogenic genes *C/ebpα*, *Pparγ*, and *aP2* (increased mRNA expression by 2‐, 2‐, and 1.5‐fold, respectively, at BPA 11.4 mg) and therefore partially through glucocorticoid receptor signaling^41^ (Table 1).

Bisphenol A's effect on inflammation and stress was also investigated recently in 6‐week‐old male obese C57Bl/6 mice that were given 50 μg/kg body weight for 3 weeks after being fed a standard or high‐fat diet for 12 weeks. In both the elevated plus‐maze test and open‐field, a high‐fat diet induced a decreased time in open arms, entries in each zone, and distance traveled. When a high‐fat diet was associated with bisphenol A, these effects were significantly exacerbated. In the elevated plus‐maze test, bisphenol A decreased the time and number of entries in open arms by 1.86‐ and 1.6‐fold, respectively, compared to a high‐fat diet alone. As for open‐field, the distance traveled, and entries in the center were also decreased by 1.2‐ and 1.5‐fold, respectively, compared to a high‐fat diet alone. Expression of the genes *Ilβ1*, *Tlr4*, *Nlrp3*, *Tnf*, and *Ccl2* involved in inflammation were also analyzed in the prefrontal cortex, amygdala, and hypothalamus. High‐fat diet significantly increased their gene expression compared to a standard diet. When associated with bisphenol A, the effect of a high‐fat diet on gene expression is exacerbated, showing an increased gene expression of *Ilβ1*, *Tlr4*, *Nlrp3*, *Tnf*, and *Ccl2* by 1.33‐, 1.5‐, 1.6‐, 2.5‐, and 3‐fold, respectively, in the prefrontal cortex. In the amygdala, gene expression of *Tnf* and *Ccl2* were increased by 1.33‐ and 1.6‐fold, respectively, and in the hypothalamus, gene expression of *Tnf*, *Ccl2*, *Tbp2*, and *Cma1* were increased by 1.33‐, 2‐, 1.75‐, and 1.25‐fold, respectively. Bisphenol A's effect was studied on the hypothalamic–pituitary–adrenal axis through analysis of the gene expression of *Crh* and its receptor *Crhr1* in the prefrontal cortex and results showed an increase in gene expression by 1.4‐ and 1.33‐fold, respectively. Overall, this study reports that bisphenol A exposure associated with diet‐induced obesity induces anxiety through hyperactivation of the hypothalamic–pituitary–adrenal axis, and stimulates the immune system in the prefrontal cortex, the amygdala, and the hypothalamus, and these effects are exacerbated when bisphenol A is associated with a high‐fat diet^42^ (Table 1).

The effect of endocrine‐disrupting chemicals on weight and social behavior in mice was also studied by Kim et al. Pregnant mice were fed the following pollutants: Tetrabromobisphenol A, 2,2,4,4‐tetrabromodiphenyl ether (BDE‐47) and bisphenol S (0.2 mg/kg/bw/day from pregnancy day 8 through postpartum day 21), and the pups were subjected to behavioral tests. The results showed no body weight difference in dams; however, the male pups' body weight was significantly decreased (1.07‐fold for BDE‐47 and 1.1‐fold for bisphenol S). Mice exposed to the pollutants moved significantly faster (1.1‐fold for BDE‐47, 1.13‐fold for bisphenol S, and 1.08‐fold for tetrabromobisphenol A) and showed rapidly decreased social interaction (1.3‐fold for BDE‐47, 1.2‐fold for bisphenol S and 1.7‐fold for tetrabromobisphenol A), suggesting increased anxiety^43^ (Table 1).

A recent study explored the endocrine‐disrupting effects of perfluorooctane sulfonic acid on the placenta of pregnant rats. Rodents were exposed to 1 and 5 mg/kg/bw/day of perfluorooctane sulfonic acid for 17 days (from gestational day 4 to 20). At gestational day 20, fetal weight decreased by 8–15% in males and 7–14% in females, and placental weight decreased by 7–14% in males and 7–11% in females. Regarding the placenta, perfluorooctane sulfonic acid did not affect the junctional zone weight, while it decreased the labyrinth zone weight by 7–12% in males and 9–14% in females. Steroid hormones were highly affected by perfluorooctane sulfonic acid, aldosterone, progesterone, testosterone, and luteinizing hormone were increased by 201%, 166%,45%, and 49%, respectively. While human chorionic gonadotropin, estradiol, and prolactin decreased by 47–62%, 27%, and 28%, respectively, and follicle‐stimulating hormone was unaffected. Perfluorooctane sulfonic acid increased corticosterone levels by 180–205%. However, corticosteroid binding globulin was decreased by 7–11%. Increased gene expression of *StAR*, *17β‐HSD1*, and *17β‐HSD3* by 1.5‐fold in females was observed while the genes *UGT1A1*, and *3β‐HSD1* were increased by 2‐ and 1.4‐fold, respectively, in males. In both sexes, *Cyp19A1* gene expression decreased by 2‐ and 2.5‐fold at 1 and 5 mg/kg/bw/day of perfluorooctane sulfonic acid, respectively, and no effect was observed on *Cyp17A1*, *SRD5A1*, and *SRD5A3* gene expression. Therefore, this study demonstrates that perfluorooctane sulfonic acid exerts an endocrine disruption on pregnant rats by affecting the placenta and inducing fetal weight reduction^44^ (Table 1).

Similar studies have also been conducted on animals other than rodents. As an illustration, the effect of PCB has also been studied on cortisol levels in sheep fetuses from polychlorinated biphenyls‐exposed mothers (98 μg/kg/bw/day of polychlorinated biphenyls 153 or 49 μg/kg/bw/day of PCB 118). This study showed lower plasma cortisol levels (1.25‐ and 1.26‐fold for each concentration, respectively), lower body weight (3.98‐ and 4.9‐fold for each concentration, respectively), and higher plasma adrenocorticotropic hormone levels (1.45‐fold for PCB 118) in highly polychlorinated biphenyls‐exposed fetuses, indicating a fetal alteration in the hypothalamic–pituitary–adrenal‐axis caused by the pollutant^45^ (Table 1).

In two separate experiments polychlorinated biphenyls and polybrominated diphenyl ethers were recently investigated on male cats (*Felis catus* from Japan). In the first experiment, 28‐month‐old cats were injected with an acute dose of 0.5 mg/kg/bw of polychlorinated biphenyls intraperitoneally and no changes in body weight were recorded, while relative testis weight was decreased by a factor of 1.33. Polychlorinated biphenyls exposure was negatively correlated with progesterone (r = −0.9429), but no effect was noted with testosterone. However, corticosterone levels were positively correlated with polychlorinated biphenyls (r = 0.8286). In the second experiment, 13‐month‐old cats were fed 7 mg/kg/bw of polybrominated diphenyl ethers for a year and, a significant increase in body weight by a factor of 1.2, relative brain weight by a factor of 1.3, relative subcutaneous fat by a factor of 2.6 and absolute subcutaneous fat by a factor of 3.2 was observed. Triglyceride levels were negatively correlated with the pollutant (r = −0.8286), and total protein and albumin levels were significantly decreased by 1.2‐ and 1.14 times, respectively. Both high‐density lipoprotein and triglyceride levels were significantly increased by a factor of 1.3 and 1.43, respectively, and mRNA gene expression of *stearoyl‐CoA desaturase (SCD)* involved in lipid metabolism was decreased by 2 times. These results show that acute exposure to polychlorinated biphenyls could disrupt the male reproductive system, corticosterone, albumin, and protein synthesis. At the same time, polybrominated diphenyl ethers induce weight and fat gain, high‐density lipoprotein, and triglycerides accumulation despite the reduction in SCD^46^ (Table 1).

##### Cellular and molecular studies

A study conducted on human adipocytes exposed to different concentrations of bisphenol A showed increased triglycerides accumulation by 1.7‐fold with 5.7 mg BPA and 2.1‐fold with 11.4 mg BPA, increased mRNA levels of the molecules associated with adipogenesis *aP2* by 3.1‐ and 3.9‐fold for each concentration of bisphenol A, respectively, *adipsin* by 1.5‐fold with 114 mg BPA, *PPARγ* by 2‐fold with 114 mg BPA, *CEBPα* by 1.8‐fold for both concentrations of bisphenol A, and *CEBPβ* by 1.5‐fold with 114 mg BPA. Bisphenol A's mechanism was studied using glucocorticoid receptor and estrogen receptor antagonists. Surprisingly, the results show that the effect of bisphenol A was suppressed by the estrogen receptor antagonist (aP2 protein levels were reduced by 75%) but not by the glucocorticoid receptor antagonist RU486, emphasizing that bisphenol A is likely to be mediated by a non‐classical estrogen receptor‐pathway and not by the glucocorticoid receptor pathway^47^ (Table 2).

Similarly, Wang et al conducted a study on preadipocytes and adipocytes collected from children (3–13 years old) undergoing abdominal surgery to assess the effect of bisphenol A on 11β‐HSD1, a glucocorticoid enzyme. In this study, adipocytes exposed to bisphenol A (2 μg, 2 mg, or 18.3 mg) for 24 h had increased *11β‐HSD1* (1.8‐,1.5‐, and 1.8‐fold, respectively), *PPARγ* (1.5‐, 1.5‐, and 3.5‐fold, respectively), and *LPL* (1.5‐, 1.5‐, and 2‐fold, respectively) mRNAs, as well as lipid accumulation (67%, 49%, and 89% of lipid droplets, respectively, compared to 45% in control). Contradictory results were shown by Wang et al regarding RU486, which is used as a glucocorticoid receptor antagonist. The effect of bisphenol A was inhibited by RU486 (1.73‐fold), suggesting that bisphenol A stimulates the activity of 11β‐HSD1 through glucocorticoid receptor signaling^48^ (Table 2).

Sargis et al studied the association between bisphenol A and the glucocorticoid pathway in 3 T3‐L1 murine preadipocyte cell lines. Bisphenol A exposure to 69 mg/l was shown to potentiate glucocorticoid receptor activity (3‐fold), and exposure to 6.9 mg/l stimulated adipocyte lipid accumulation (6.2‐fold), thus promoting adipocyte differentiation^49^ (Table 2).

The importance of glucocorticoid receptor signaling on bisphenol A activity was also studied through 3 T3‐L1 cells exposed to bisphenol A without exogenous glucocorticoids. Upregulation of adipsin (2‐fold increase at 0.2 and 2 μg of bisphenol A) and the adipogenic marker aP2 (or fatty acid binding protein 4) (2.5‐fold increase at 2 μg of bisphenol A) were observed with or without exogenous glucocorticoids. Atlas et al demonstrated that glucocorticoids are not necessary to allow cell differentiation. Moreover, transfected Cos‐7 cells, which do not express endogenous glucocorticoid receptors, were exposed to 5.7 mg of bisphenol A for 24 h, revealing that bisphenol A promotes glucocorticoid receptor activity by a factor of 3, especially in the presence of C/EBPδ (4‐fold). Bisphenol A may, therefore, affect the upregulation of aP2 through the recruitment of a transcriptional complex containing glucocorticoid receptor and C/EBPδ on the aP2 promoter^50^ (Table 2).

Tetrabromobisphenol A, one of the most commonly used flame retardants, was also studied to differentiate 3 T3‐L1 cells. Preadipocytes were exposed to tetrabromobisphenol A (from 0.8 to 17.9 μg in 0.2 ml adipocyte maintenance media), and results highlighted a significantly increased number of adipocytes (between 1‐ and 1.5‐fold). mRNA expression of the genes *Pref1* and *Thy1*, involved in adipogenesis repression, was significantly decreased by tetrabromobisphenol A (1.12 and 1.67‐fold, respectively), while the genes *C/EBPδ*, *SREBF1*, *FKBP5*, and *KLF15*, involved in adipocyte differentiation, were significantly increased by 1.2‐, 1.5‐, 1.05‐, and 1.5‐fold, respectively. PPARγ receptor was weakly activated by tetrabromobisphenol A in a dose‐dependent manner (by 4.07‐fold with 5.4 μg of tetrabromobisphenol A and 12.11‐fold with 17.9 μg of tetrabromobisphenol A), suggesting a partial agonism of PPARγ by tetrabromobisphenol A. Glucocorticoid signaling was also investigated due to its association with C/EBPδ, FKBP5, and KLF15, so as to study glucocorticoid receptor‐mediated adipocyte differentiation. A low concentration of tetrabromobisphenol A induced a significant increase in glucocorticoid receptor mRNA expression by 1.25‐fold. TBBPA exposure resulted in elevated protein concentrations of aP2 (by 5.56‐fold), adiponectin (by 3‐fold), leptin (by 2‐fold), fatty acid synthase (Fasn) (by 3‐fold), and LPL (by 2.75‐fold). The expression of the insulin receptor and its adaptor protein were both increased by tetrabromobisphenol A as well by 2‐ and 1.5‐fold, respectively^51^ (Table 2).

Liu *et al* assessed the binding affinity of tetrabromobisphenol A for PPARγ and glucocorticoid receptors in 3 T3‐L1 cells. They demonstrated that tetrabromobisphenol A and its analogs had a significantly increased transactivation of PPARγ (with an EC_20_ of 7.7 ± 1.1 μM) and a high binding affinity for PPARγ (with an EC_20_ of 5.7 ± 0.9 μM) in a dose‐dependent manner (for 0.5–27 mg of tetrabromobisphenol A). Similar results indicated tetrabromobisphenol A's ability to bind to glucocorticoid receptors (with an EC_20_ of 0.5 ± 0.1 μM for 0.5–5.4 mg of tetrabromobisphenol A). Interestingly, some tetrabromobisphenol A agonists had greater binding affinity for PPARγ (with a higher EC_20_ of 2‐ to 3‐fold) and glucocorticoid receptor (with a higher EC_20_ of 20‐ to 200‐fold) than tetrabromobisphenol A. After confirming tetrabromobisphenol A's affinity for PPARγ and glucocorticoid receptor, Liu *et al* investigated the effect of the pollutant on adipocyte differentiation. They observed significant triglycerides accumulation (by 3‐fold for tetrabromobisphenol A and 4.8‐fold for its analog at 10.9 mg) upholding the effect of tetrabromobisphenol A as an adipogenic differentiation promotor. The concentration and mRNA expression of adiponectin, perilipin, *PPARγ*, and *FABP4* were all significantly increased (by 113‐, 170.8‐, 112.2‐, and 79.9‐fold, respectively) by tetrabromobisphenol A and its analogs. These findings suggest that tetrabromobisphenol A and its analogs induce adipogenic effects that are partially mediated by the activation of PPARγ and glucocorticoid receptor signaling^52^ (Table 2).

Plastic products containing endocrine disrupting chemicals (triphenyl phosphate, diphenyl phosphate, tert‐butylphenyl diphenyl phosphate (BPDP), 2‐ethylhexyl diphenyl phosphate (EHDP), tri‐o‐cresyl phosphate (TOCP), tris (2 butoxyethyl) phosphate (TBEP), allethrin, DEHP, dibutyl phthalate, di‐iso‐nonyl phthalate (DINP), and benzyl butyl phthalate) were used to investigate their adipogenic activity in 3 T3‐L1 preadipocytes. They showed that plastics containing EHDP and triphenyl phosphate increased adipocyte differentiation by a factor of 1.4, plastics containing diphenyl phosphate and triphenyl phosphate increased it by a factor of 1.9, and plastics containing TOCP, DINP, TBEP, benzyl butyl phthalate, dibutyl phthalate, and BPDP increased adipogenesis by a factor of 1.8. Similarly, all chemicals except plastics containing the combined flame retardants triphenyl phosphate and EHDP, increased lipid droplet area per adipocytes by 21.6–114%. Triphenyl phosphate increased PPARγ activity by 7.3–34,7%, and when triphenyl phosphate is associated with diphenyl phosphate the activity is increased by 10.3–24.4%. Interestingly, no endocrine‐disrupting chemical was able to stimulate glucocorticoid receptor activity. These data suggest that adipocyte differentiation induced by the flame retardants is not necessarily mediated by the PPARγ signaling pathway and is unlikely to be mediated via glucocorticoid receptor signaling. Of note, this study did not report precise concentrations of flame retardants^53^ (Table 2).

### 3.4 Pesticides

#### 3.4.1 Herbicides

##### Animal studies

Among herbicides, a study was conducted on nonylphenol, a non‐ionic surfactant widely used in household products, in which pregnant female rats (2‐month‐old) were given nonylphenol (2 μg/ml) in drinking water during gestation (approximatively 400 μg/ml/day) and lactation (approximatively 800 μg/ml/day). In male offspring, results showed an increased body weight (1.2‐fold), as well as increased plasma levels of adrenocorticotropic hormone, corticosterone, aldosterone, and aldosterone synthase activity (51%, 600%, 177%, and 77.1%, respectively). The expression of 11β‐HSD1 was increased by 50%, and its activity was increased in the liver (by 29.3%), adipose tissue (by 25.5%), and adrenal cortex (by 91.6%). The expression of the steroidogenic acute regulatory (StAR) protein was significantly increased by 50%. These results suggest that nonylphenol alters the hypothalamic–pituitary–adrenal axis through negative feedback of corticoids, leading to an increased adrenocorticotropic hormone concentration. In female offspring, plasma corticosterone (by 105%), plasma aldosterone (by 74%), and 11β‐HSD1 activity in the liver (by 31%) and adipose tissue (by 20%) were significantly increased. Thus, nonylphenol induced aldosterone increase in female offspring but not adrenocorticotropic hormone, suggesting that nonylphenol may be mediated by the renin‐angiotensin system, leading to hypertension. Chang et al suggest that steroid hormones may also play a key role in the differences observed between male and female offspring^54^ (Table 3).

**TABLE 3:** In vivo studies recounting the effects of pesticides.

Type of pesticides	Pollutants and concentrations	Species/age/ethnicity	Results	References
Herbicides	Nonylphenol: 400 μg/kg/day during gestation and 800 μg/kg/day during lactation	Animal: 2‐months old pregnant rats (Sprague–Dawley)	NP increases aldosterone, corticosterone levels, and 11β‐HSD1 activity NP's effect depends on sex NP increases plasma ACTH and adrenal corticoid in males NP increases body weight in males NP did not affect ACTH in females	Chang et al, 2012
Herbicides	Atrazine: 10 mg/kg (1dose, orally)	Animal: 60 days‐old pregnant mice (C57Bl/6 J)	Atrazine alone did not affect body weight and glucose tolerance When mixed with PFOA, BPA, and atrazine, treatment for 12 days induced maternal glucose tolerance alteration	Merrill et al, 2021
Herbicides	Glyphosate: 50 mg/kg/day for 3 weeks	Animal: Pubertal (21‐days‐old C57Bl/6 J) mice	Glyphosate exacerbates the obesogenic effects of high‐fat diet, especially in females Glyphosate‐induced increased cortisol plasma levels	Rosolen et al, 2024
Herbicides	Atrazine: 200 and 400 μg/l for 14 days	Animal: *Xenopus laevis* tadpoles	Atrazine increased the expression of genes involved in ketone bodies metabolism, autophagy, urea cycle, glutathione metabolism, citric acid cycle, and amino acid metabolism Atrazine decreased the expression of genes involved in fat storage, fatty acid elongation, gluconeogenesis, glycolysis, carbohydrate metabolism Atrazine increased PPARβ activity but decreased GR activity	Zaya et al, 2011
Herbicides	Glyphosate: 12000 mg/l for 3 months	Animal: Argentine tegu	Pollutants induced high levels of plasma corticosterone, and immunosuppression Plasma corticosterone level is negatively correlated with body mass	Mestre et al, 2019
Insecticides	Methamidophos: 2 μg/kg for 15 days	Animal: Male swiss mice (90 days old)	MTP and alteration of the circadian rhythm affect the reproductive system and steroidogenesis MTP did not affect corticosterone levels	Maia et al, 2011
Insecticides	Mirex: 40 or 20,000 mg/l, IP for 4 days	Animal: 6–8‐year‐old rats (Wistar)	Mirex induces hypoglycemia and hyperlipidemia Mirex inhibits the activity of adrenal corticosteroid‐synthesizing enzymes Mirex does not affect β‐cells	Jovanovich et al, 1987
Insecticides	Cypermethrin (CPF) 2 mg/l, Chlorpyrifos (CYP) 0.01 mg/l for 3 months	Animal: Argentine tegu	Pollutants induced high levels of plasma corticosterone, and immunosuppression Plasma corticosterone level is negatively correlated with body mass	Mestre et al, 2019
Insecticides	Pyriproxyfen and spirodiclofen: single dose of 21, 42 or 84 ng orally	Animal: Bees (*apis mellifera*)	Insecticides stimulate abdominal lipid Insecticides induced stress‐like response in bees represented by foraging alteration	Deeter et al, 2023
Fungicides	Detection of HCB: 21.1 to 36.4 ng/g lw	Human: Obese Norwegian patients (18–60 years old)	Increased blood level of HCB after bariatric surgery HCB increase is associated with serum cortisol reduction HCB affects HPT‐ and HPG‐axes HCB affects testosterone in males	Jansen et al, 2020
Fungicides	TF: 100 mg/kg diet for 12 weeks	Animal: 8‐week‐old C57BL/6 male mice	TF increases fat, body weight, and adipose tissue TF reduces genes involved in fatty acid oxidation TF activates GR and tends to activate PPARγ TF induces insulin resistance	Regnier et al, 2015
Fungicides	TF: 100 mg/kg diet for 16 weeks	Animal: FS‐Mpc2^−/−^ mice	TF had no effect on adiposity, body weight, adipose tissue gene expression, and other metabolic parameters	Chen et al, 2018
Fungicides	TBT: 25 μg/kg; p,p’‐DDT: 2 mg/kg for 10 months IP	Animal: LepR‐Cre male mice (4–6 weeks old)	TBT‐induced anxiety and weight gain p,p’‐DDT reduced locomotion Fungicides exert obesogenic effect through hypothalamic circuit controlling energy metabolism	Yavuz et al, 2023
Fungicides	TBT: 10 ng/kg/day for 15 or 30 days	Animal: Adult female Wistar rat (12 weeks old)	TBT alters the HPA‐axis TBT induces increased plasma corticosterone levels, cholesterol levels, lipid accumulation, PPARγ protein expression, inflammation, apoptosis TBT causes adrenal cortex hypertrophy	Merlo et al, 2016
Fungicides	TBT: 10 to 50 ng/l for 9 months	Animal: Zebrafish (*Danio rerio*)	TBT increases body weight in males, adipose mass, triglycerides TBT causes hepatic metabolic dysfunction TBT's effect is more powerful at low doses TBT acts through the PPARγ and RXR signaling pathways TBT affects the liver and the brain	Lyssimachou et al, 2015

Abbreviations: 11β‐HSD1, 11β‐Hydroxysteroid dehydrogenase type 1; ACTH, adrenocorticotropin hormone; BPA, Bisphenol A; CPF, Cypermethrin; CYF, Chlorpyrifos; DDT, dichlorodiphenyltrichloroethane; GR, glucocorticoid receptor; HCB, hexachlorobenzene; HCH, Hexachlorocyclohexane; HPA, hypothalamic–pituitary–adrenal; HPG, hypothalamic pituitary‐gonadal; HPT, hypothalamic pituitary‐thyroid; IP, intraperitoneal; lw, lipid weight; MTP, Methamidophos; NP, Nonylphenol; PFOA, perfluorooctanoic acid; PPAR, Peroxisome Proliferator‐activated Receptor; RXR, retinoid X receptor; TBT, Tributyltin; TF, Tolyfluanid.

The ability of glyphosate to induce obesity was assessed in pubertal C57Bl/6 mice (21 days old) exposed to 50 mg/kg/day for 3 weeks. Results revealed that high‐fat diet‐fed mice exposed to the herbicide showed a 48% increase in body fat content, a 3.2‐fold increase in body weight, a 19% increase in food intake, and a 31% decrease in lean mass in females. Adipocytes were hypertrophied. In females, an 18% increase in perigonadal white adipose tissue weight, a 16% increase in size, and a 24% increase in retroperitoneal white adipose tissue size were observed. In comparison, in males, a 41% increase in size and a 31% increase in retroperitoneal white adipose tissue size were noted. The hormone‐sensitive lipase protein content in perigonadal white adipose tissue was increased in female mice exposed to the herbicide whether it was fed a high‐fat diet (by 188%) or a standard chow diet (by 136%). Glyphosate had no effect on brown adipose tissue weight, however, a 28% increase in brown adipose tissue and a 34% increase in lipid vacuole were observed in female's brown adipocytes. Regarding plasma cortisol, a high‐fat diet alone did not affect cortisol concentrations while glyphosate exposition increased cortisol levels by 80% in males and by 180% in females. These data provide evidence that pubertal glyphosate exposure aggravates the obesogenic effects of a high‐fat diet in adulthood, especially in females, which may be mediated by altered levels of sexual hormones. As the pollutant induces stress, the authors suggest that this factor may also account for obesity^55^ (Table 3).

Zaya et al assessed the link between the herbicide atrazine and metabolic pathways in *Xenopus laevis* tadpoles by exposing them to either 200 or 400 μg/ml of atrazine in glass bowls containing 1 L of water for 2 weeks. Results showed increased mRNA expression of genes involved in the following pathways: ketone body metabolism (Z‐score = 4.31), glutathione metabolism (Z‐score = 2.44), citric acid cycle (Z‐score = 2.44), urea cycle (Z‐score = 3.97), amino acid metabolism (Z‐score = 2.51–3.86), and regulation of autophagy (Z‐score = 2.74). Conversely, the down‐regulated pathways identified were the glycolysis and gluconeogenesis pathway (Z‐score = 2.31), the fatty acid elongation pathway (Z‐score = 3.61), and the ribosomal structural protein pathway (Z‐score = −2.40). These results suggest that tadpoles catabolize proteins to produce energy. Additionally, the biochemical analysis demonstrated a significant decrease in acyl‐CoA and glucocorticoid receptor expression (68% and 66%, respectively, at 400 μg/ml)^56^ (Table 3).

Mestre et al conducted an experimental study on the effects of the glyphosate herbicide on the endocrine and immune system of the Argentine tegu. These reptiles were sprayed with a pesticide mixture containing 66.2% of glyphosate (12,000 mg/l) and the following insecticides: 25% of cypermethrin (representing 0.01 mg/l) and 48% of chlorpyrifos (representing 2 mg/l) in sub‐chronic conditions for 3 months. Results showed elevated plasma corticosterone and increased number of lobes in heterophils (1.45‐ and 1.6‐fold, respectively), and fewer leucocytes (by 1.13‐fold) and natural antibodies titers from lizard plasma (by 1.3‐fold). A trend toward decreased body weight was also noted (1.16‐fold). This study therefore confirms an alteration in immunological parameters due to the toxic effects of glyphosate and suggests that the endocrine system might mediate its effects^57^ (Table 3).

##### Cellular and molecular studies

Several herbicides were tested on 3 T3‐L1 adipocytes with different concentrations ranging from 0.4 to 373 mg (in 100 ml maintenance medium), as follows: Quizalofop‐p‐Ethyl (QpE), glyphosate, 2, 4‐D, dicamba, isoxaflutole, mesotrione, quizalofop acid, popaquizafop, tetrahydrofurfuryl alcohol, and 2, 3‐dihydroxyquinoxaline. Lipid accumulation was significantly increased when cells were treated with isoxaflutole (2 times for concentrations of 18 and 36 mg), dicamba (2.5‐fold for 111 mg), and QpE (in a dose‐dependent manner, starting with 1.9 mg and reaching a 3‐fold increase with concentrations between 18.6 and 37.3 mg). QpE did not alter the adipogenic genes *LPL*, *aP2*, and *Adipoq*. However, QpE proved to be involved in altering the “nuclear receptors in lipid metabolism and toxicity” pathway. The affinity of QpE (for 37.3 mg) to bind to PPARγ was increased 2 times, and neither the antagonists of glucocorticoid receptor (RU486) nor estrogen receptor were capable of blocking the lipid accumulation mediated by QpE. These findings suggest that QpE induces lipid accumulation through partial activation of the PPARγ signaling^58^ (Table 4).

**TABLE 4:** In vitro studies recounting the effects of pesticides.

Type of pesticides	Pollutants and concentrations	Cell type	Results	References
Herbicides	QpE, Glyphosate, 2, 4‐D, Dicamba, Isoxaflutole, Mesotrione, Quizalofop acid, Popaquizafop, Tetrahydrofurfuryl alcohol, 2, 3‐dihydroxyquinoxaline: 0.1 to 1000 μM	3 T3‐L1	Dicamba and isoxaflutole exert adipogenic effects QpE increases lipid accumulation partially through the activation of PPARγ	Biserni et al, 2019
Insecticides	Endrin: 100 nmol/l and 1 μmol/l for 3 days	3 T3‐L1	Endrin stimulates GR activity Endrin stimulates adipogenesis and lipid accumulation through the GR pathway	Sargis et al, 2010
Fungicides	TBT, TF and TPT: 100 nmol/l and 1 μmol/l for 3 days	3 T3‐L1	TF stimulates GR activity, adipogenesis, and lipid accumulation TPT stimulates PPARγ activity but does not activate GR	Sargis et al, 2010
Fungicides	TF: 1 nM to 1 μM for 48 h	Primary murine adipocyte (from 8 weeks‐old male C57BL/6 mice)	TF activates GR, induces genes involved in lipid synthesis, insulin‐stimulated lipogenesis, and increases IRS‐1 gene expression	Neel et al, 2013
Fungicides	TF, TBT: 0.1 to 25 μM for 3 days	BAT, Adipocyte from mice with deletion of MPC, MEFs, MDA‐kb2 cells	TBT induced a drop in pyruvate respiration TF increased MPC gene and protein expression TF did not activate GR or affect GR phosphorylation TF had no effect on obesity	Chen et al, 2018
Fungicides	MBT, DBT, TBT, TeBT, MPT, DPT, TPT, SnCl4: 10 or 50 ng/ml for 10 days	3 T3‐L1 preadipocytes	TBT, TPT, and DBT stimulate adipogenesis TPT and DBT may interfere with TBT's effect on adipocyte differentiation through PPARγ pathway The fungicides do not act through the GR pathway	Ticiani et al, 2023

Abbreviations: 3 T3‐L1, mouse embryonic fibroblasts; AhR, aryl hydrocarbon receptor; AR, androgen receptor; BAT, Brown adipose tissue from female mice; CALUX, rat hepatoma cells; DBT, bibutyltin; DPT, diphenyltin; ER, Estrogen receptor; GR, glucocorticoid receptor; IRS‐1, insulin receptor substrate‐1; MBT, monobutyltin; MEFs, mouse embryonic fibroblasts; MDA‐kb2, human breast cancer cells; MPC, mitochondrial pyruvate carrier; MPT, monophenyltin; MVLN, Human breast carcinoma cells; PPARγ, peroxisome proliferator–activated receptor; QpE, Quizalofop‐p‐Ethyl; SnCl_4_, tin tetrachloride; TBT, Tributyltin; TeBT, tetrabutyltin; TF, Tolylfluanid; TPT, triphenyltin.

#### 3.4.2 Insecticides

##### Animal studies

The effects of the organophosphate methamidophos (MTP) on the mice's reproductive system were assessed in males who were exposed to 0.002 mg/kg bw of MTP in their drinking water for a 15‐day period. A slight weight loss was observed (−0.02 g). Testosterone levels were significantly decreased by six times, while progesterone levels increased by eight times. A 40% decrease in seminal vesicle and a 58% decrease in epididymal weight were additionally observed. Interestingly, the treatment did not affect corticosterone levels, suggesting that the MTP's deleterious effects on the males' reproductive system may not be mediated by stress^59^ (Table 3).

A recent study conducted by Deeter et al investigated the relationship between pollutant‐induced stress, behavior, and fat storage. Adult *Apis mellifera* bees were subjected to a single oral dose of either low (21 ng), medium (42 ng), or high (84 ng) amounts of the insecticides pyriproxyfen and spirodiclofen. The mortality rate in bees increased by 50% at day 17 after pyriproxyfen exposure, and the authors found that exposed bees started foraging earlier than control bees by 1.2‐fold. However, no effect was observed on abdominal lipids. As for spirodiclofen, no effect on mortality was reported. A reduction in total abdominal lipids by 1.1‐fold was recorded, followed by a decrease in pollen collected, which contained a significantly higher lipid concentration than in pollen collected by controls (by 1.13‐fold). These results suggest that the stress response induced by the insecticides can exert long‐lasting effects on lipid homeostasis and storage^60^ (Table 3).

##### Cellular and molecular studies

Sargis et al looked at the effect of 38.1 and 381 μg/l of endrin on 3 T3‐L1 cells. Endrin stimulated lipid accumulation by 6.4 times and potentiated glucocorticoid receptor activity by 3.25 times, thus promoting adipocyte differentiation^49^ (Table 4).

#### 3.4.3 Fungicides

##### Human studies

Jansen et al looked at the effects of the fungicide hexachlorobenzene (21.1 to 36.4 ng/g lw) on thyroidal hormones, and the reproductive and endocrine systems in people with obesity. A positive association was demonstrated between hexachlorobenzene and serum testosterone level (B 95% CI: 0.0146, p = 0.016). A negative trend between hexachlorobenzene and cortisol (B 95% CI: −1.394, p = 0.059) was observed, suggesting that hexachlorobenzene may act on both the hypothalamic–pituitary‐gonadal‐ and hypothalamic–pituitary‐thyroid‐axes and may be considered as an anti‐glucocorticoid^39^ (Table 3).

##### Animal studies

The effect of tolylfluanid, the most used fungicide in European agriculture, was assessed in male mice supplemented with 1 mg/l of tolylfluanid for 12 weeks. An increased food consumption by 1.5 times and a 19% weight gain were noticed. Adipose tissue was significantly increased by 28% with a 1.18‐fold reduction in the number of hypertrophied adipocytes. Tolylfluanid significantly increased leptin levels by 1.6 times and decreased adiponectin levels by 31%, inducing an increased leptin/adiponectin ratio of 67%, a well‐known biomarker of metabolic dysfunction. These mice also exhibited insulin and glucose resistance (1.14‐ and 1.12‐fold, respectively) with a reduced hormone‐sensitive lipase by 1.33 times, reduced gene expression of the insulin receptor substrate‐1 (IRS‐1) by 30%, and a decreased insulin‐stimulated Akt phosphorylation by 1.9 times. A 1.05‐fold decrease in respiratory exchange ratio, a 1.15‐fold decrease in body‐mass energy expenditure, and a 1.33‐fold decrease in genes involved in fatty acid oxidation were observed, pointing towards an increased activity and energy expenditure by 1.135 and 1.03 times, respectively. The ability to shift between fuel sources was altered by 47%, suggesting a strong metabolic inflexibility mediated by tolylfluanid. Glucocorticoid receptor‐dependent gene expression pattern showed an overall up‐regulation (p < 0.001, 533 unique genes), suggesting energy modulation mediated through glucocorticoid receptor signaling activation. In summary, this study investigates the obesogenic effect of tolylfluanid through altered adipose tissue and metabolic health disruption and suggests that tolylfluanid may possess a potential glucocorticoid effect^61^ (Table 3).

A recent study explored the effects of dichlorodiphenyltrichloroethane (p,p’‐DDT) and tributyltin on the electrical activity of dorsomedial hypothalamic leptin receptor neurons in LepR‐Cre male mice aged between 4 and 6 weeks. Mice were administered 2 mg/kg of p,p’‐DDT, or 25 μg/kg of tributyltin intraperitoneally for 10 months. While no significant change in food intake was observed, body weight tended to increase in the mice treated with tributyltin (by 1.2 times, p = 0.054). The mice's behavior was assessed through the open‐field test. A 2‐fold reduction in time spent in the center was reported for the mice treated with tributyltin. In addition, a 2.75‐ and 1.83‐fold decrease in total distance traveled by the mice treated with tributyltin and p,p’‐DDT were observed, respectively. A 4‐fold decrease in spontaneous inhibitory postsynaptic current frequency was observed with both fungicides. A 4.6‐fold increase in c‐Fos activity with tributyltin and a 6‐fold increase with p,p’‐DDT were also shown. These data suggest that fungicides, and especially tributyltin, act as an obesogen and induce anxiety through hypothalamic circuits controlling energy metabolism^62^ (Table 3).

The effects of tributyltin on the hypothalamic–pituitary–adrenal‐axis were assessed on female rats administered 10 ng/kg/day of tributyltin by gavage for 15 and 30 days. Pituitary gland weight was increased by 14% in rats exposed for 15 days and 37% in those exposed for 30 days. Mitosis, hyperplasia, and disorganization of cell cords were also impacted in the pituitary gland. Adrenocorticotropic hormone expression was significantly decreased by 1.2 times in the rats exposed for 15 days, and by 1.3 times in those exposed for 30 days. N‐acetyl‐β‐d‐glucosaminidase activity (by 2.55‐fold), myeloperoxidase activity (by 1.73‐fold), and the number of mast cells (by 1.45‐fold) involved in the inflammatory and immune system were all significantly increased in the rats exposed to tributyltin for 30 days. Collagen surface density increased by 1.3 times in the rats exposed to tributyltin, and both the reactive oxygen species O^2−^ (1.74‐ and 1.43‐fold in rats exposed for 15 and 30 days, respectively), and the apoptosis marker caspase 3 (1.22‐ and 1.35‐fold in rats exposed for 15 and 30 days, respectively) were increased as well. Adrenal cortex analyses showed mitotic activity, hyperplasia, and a 15% increase in the cortical area. StAR expression increased by 1.5 and 2.5 times in the rats exposed for 15 and 30 days, respectively, and PPARγ expression increased by 1.25 and 1.36 times in the rats exposed for 15 and 30 days, whereas the expression of CYP_11B_ was unchanged. An increased N‐acetyl‐β‐d‐glucosaminidase activity (1.42‐fold), myeloperoxidase activity (3.2‐fold), and the number of mast cells (1.33‐fold) in adrenals was observed in the rats exposed for 30 days. Collagen density, adrenal cholesterol, and lipid accumulation were also significantly increased (by 2.8, 1.96, and 1.4 times in the rats exposed for 30 days). Increased reactive oxygen species production (by 2.56 and 4.22 times in the rats exposed for 15 and 30 days, respectively) and markers of apoptosis (by 1.3 and 1.5 times in rats exposed for 15 and 30 days, respectively) were reported. Adrenocorticotropic hormone adrenal plasma increased by 2.14‐fold and 1.76‐fold in rats exposed for 15 and 30 days, respectively. Analyses were also carried out on the rats with bilateral adrenalectomy (ADX) and showed an exacerbated plasma adrenocorticotropic hormone plasma concentration increase by a factor of 7.26 and 4.86 in the rats exposed for 15 and 30 days, respectively, as compared to controls. A 1.28‐fold increase in inducible nitric oxide synthase and a 70 to 90% increase in corticotropin‐releasing hormone (CRH) was observed in the hypothalamus. The basal levels of corticosterone (3‐fold), adrenocorticotropic hormone (2.3‐fold), and cholesterol (1.96‐fold) were significantly increased in the rats exposed to tributyltin for 30 days. Altogether, these findings suggest that tributyltin alters the hypothalamic–pituitary–adrenal‐axis in female rats by inducing lipid accumulation in adrenals, inflammation, and steroidogenesis in both the adrenal cortex and pituitary gland, possibly through PPARγ signaling pathway^63^ (Table 3).

The effects of tributyltin were investigated on zebrafish exposed to 10 to 50 ng/l of tributyltin from the pre‐hatch stage and for 9 months. Tributyltin exposure at 10 ng/l resulted in a 10% increase in body weight in male fish and an 11% increase in the condition factor (also known as the body‐mass index in humans) in both male and female zebrafish. A 2.2‐fold increase in triglyceride levels was observed in males at 10 ng/l of tributyltin, while an increased hepatosomatic index by 33–40%, reflecting hepatomegaly, was observed in females. The *DGAT2, PPARγ, C/EBPα, C/EBPβ*, and *RXRα/a* gene expression profiles involved in adipogenesis were analyzed in the brain and liver. In the brain, a decreased mRNA level of *DGAT2* by 59% in males and 58% in females at 10 ng/l of tributyltin was observed, while at 50 ng/l of tributyltin, *DGAT2* was reduced by 64% in both sexes. At 10 ng/l of tributyltin, *C/EBPα*, and *C/EBPβ* expression was decreased by 30% in males and 1.2 times in females, and RXRα/a was decreased by 45% in males and 43% in females. As for the liver, *DGAT2* and *C/EBPβ* were decreased by 43% in males, and *C/EBPα* was decreased by 30% at 10 ng/l of tributyltin, while *PPARγ* was increased by 34% in males and 25% in females. The *ACOX1, ACCα, ChREBP, SREBP1*, and *FASn* genes, involved in lipogenesis, were also analyzed in the brain and liver. Regarding the brain, exposure to 10 ng/l of tributyltin induced a decrease of 26% in *ACOX1* and of 47% in *ACCα* in females, while *ChREBP* and *FASn* were decreased in males by 20% and 27%, respectively. *SREBP1* was increased by 39% in males at 50 ng/l of tributyltin. Analysis of the *11β‐HSD2, 11β‐HSD3α, IGF‐Iα*, and *IGF‐IIα* genes, involved in the glucocorticoid metabolism, revealed an up‐regulation of *11β‐HSD2* by 2 times (at 50 ng/l of tributyltin), and a downregulation by 45% of *IGF‐IIα* in the brain. Conversely, for the liver, a decreased regulation of *11β‐HSD2* by 48% at 50 ng/l of tributyltin and upregulation of *IGF‐IIα* by a factor of 3 in males and 2 in females were noted. The expression profiles of the remaining genes involved in the glucocorticoid metabolism were not significantly impacted by tributyltin exposure. These findings point out that chronic exposure to the obesogen tributyltin disrupts the lipid metabolism in both the brain and liver in a sex‐dependent manner, especially at low concentrations. The effect of tributyltin seems to be mediated by the glucocorticoid receptor, PPARγ, and RXR signaling pathways^64^ (Table 3).

##### Cellular and molecular studies

Sargis et al assessed the effect of the fungicides tolylfluanid (at 35 μg and 0.35 mg/l) and triphenyltin (at 39 μg and 0.39 mg/l) on 3 T3‐L1 adipocytes. A 6.4‐fold and 3‐fold increase in lipid accumulation and glucocorticoid receptor activity, respectively, was observed in 3 T3‐L1 cells following tolylfluanid exposure. As for triphenyltin, a 2.75‐fold increase in PPARγ activity was observed. These findings suggest that while tolylfluanid may act through glucocorticoid receptor signaling, triphenyltin seems to act through the PPARγ pathway^49^ (Table 4).

The effect of tolylfluanid was investigated on primary murine adipocyte at a concentration of 35 μg/l. A 119% increase in the presence of pS220, a 131% increase in glucocorticoid receptors in the nucleus, and a 40% decrease in glucocorticoid receptor's cytoplasmic expression were induced by tolylfluanid. Additionally, a 48 to 81% increase in the glucocorticoid receptor activity and a 2.1‐fold increase in glucocorticoid receptor's binding affinity to glucocorticoid response element were observed. Tolylfluanid significantly increased IRS‐1 expression by 85%, which decreased by 1.33 after 24 h of exposure. The expression of the *HK2*, *SCD1*, and *ACC* genes, involved in lipogenesis significantly increased by 2.4, 1.6, and 2.5 times, respectively, while FAS gene expression remained unchanged. A 36% increase in insulin‐stimulated dephosphorylation of ACC, 35% in insulin‐stimulated lipogenesis, and 96% in T308 phosphorylation of Akt were also observed. These findings suggest that tolylfluanid acts as an endogenous glucocorticoid receptor agonist and promotes lipogenesis by modulating IRS‐1 expression through glucocorticoid receptor signaling^65^ (Table 4).

Chen *et al* investigated the effects of tolylfluanid and tributyltin on brown adipose tissue from female mice, adipocytes from mice with mitochondrial pyruvate carrier (MPC) deletion, mouse embryonic fibroblasts and human breast cancer cells (MDA‐kb2). These cells were exposed to different concentrations of the fungicides ranging from 29 μg to 8.7 mg/l. In brown adipose tissue, tolylfluanid reduced both the succinate‐mediated respiration (oxygen consumption of 30%) and the pyruvate‐mediated respiration at 8.7 mg/l (oxygen consumption of 17%), while tributyltin suppressed the pyruvate respiration completely, but not the succinate‐mediated respiration. Tributyltin, unlike tolylfluanid, also reduced the pyruvate respiration in MPC^−/−^ adipocytes by 2.4 times, suggesting that tolylfluanid requires MPC2 to affect mitochondrial pyruvate metabolism. Regarding mouse embryonic fibroblasts, acute exposure to tolylfluanid at 0.35 mg/l affected neither glucocorticoid receptor target genes (*GILZ, Lcn2, Lpin, PDK4, Acadm*) nor triglyceride accumulation. While exposure to tolylfluanid for 3 days led to a 1.8‐fold increase in *Lpin1* expression (at 3.5 and 35 μg/l), 1.8‐fold increase in *Stearoyl‐CoA Desaturase (SCD‐1)* expression (at 350 μg/l), 1.5‐fold increase in *FASn* expression (at 350 μg/l), and a decreased gene expression of adiponectin (*Adipoq)* by a factor of 2 (at 0.35 and 35 μg/l). As for MDA‐kb2, exposure to tolylfluanid showed no effect on glucocorticoid receptor activity or glucocorticoid receptor phosphorylation, suggesting that tolylfluanid may stimulate the genes involved in adipogenesis without using the glucocorticoid receptor signaling^66^ (Table 4).

The effect of fungicides (monophenyltin, diphenyltin, triphenyltin, monobutylin, dibutylin, tributylin, tetrabutylin, and tin chloride) on adipocyte differentiation was assessed on 3 T3‐L1 preadipocytes at 10 ng/ml or 50 mg/ml for 10 days. The results show that only dibutylin (by 1–2%), tributyltin (by 8–13%), and triphenyltin (by 5–17%) increased lipid accumulation in a dose‐dependent manner. When the fungicides are combined, lipid accumulation is less efficient, with an increased accumulation of 10% and 3.5% at 10 ng/ml and 50 mg/ml, respectively, compared to control. When combined with the glucocorticoid receptor agonist dexamethasone, dibutylin, tributyltin, and triphenyltin did not affect the effect of dexamethasone on lipid accumulation. However, when combined with the PPARγ agonist rosiglitazone, dibutylin, and triphenyltin at 50 mg/ml induced a 30% decrease in adipogenic differentiation. Similarly, dibutylin increased *aP2, PPARγ*, and *ADIPOQ* gene expressions by 3, 2.5, and 2.5 times, and tributyltin increased *aP2, PPARγ*, and *ADIPOQ* gene expressions by 20, 5, and 5 times. At 10 ng/ml, triphenyltin increased *aP2*, *PPARγ*, and *ADIPOQ* gene expressions by 25, 5, and 9 times, while at 50 ng/ml *aP2, PPARγ*, and *ADIPOQ* gene expressions increased by 45, 9, and 12 times. When the fungicides are combined, a less efficient increase in gene expression of *aP2* (by 25 times at 10 ng/ml and 2.5 times at 50 ng/ml) and *PPARγ* (by 5 times at 10 ng/ml and 2 times at 50 ng/ml) were observed. The combined chemicals induced a 5‐fold increase in *ADIPOQ* gene expression at 10 ng/ml. At 50 ng/ml, a 5‐fold decrease in gene expression was noted. When dibutylin, tributyltin, and triphenyltin were combined with dexamethasone, the effect of the agonist on *PPARγ* and *ADIPOQ* gene expression was significantly decreased by a factor of 4 and 2, respectively, while the effect of dexamethasone combined with triphenyltin at 50 ng/ml was increased by a factor of 1.5. As for the PPARγ agonist, the effect of rosiglitazone on *aP2* gene expression was decreased by 3.14 times when combined with dibutylin, and increased by 1.27 times when combined with triphenyltin at 50 ng/ml. The effect of PPARγ agonist on *PPARγ* gene expression was decreased by 7 and 2 times, respectively when combined with dibutylin and triphenyltin, respectively, and both fungicides induced a 4‐fold decrease in mRNA expression of *ADIPOQ* at 50 ng/ml. Dibutylin, tributyltin, and triphenyltin separately stimulate adipocyte differentiation, but not when combined. The authors also suggest that among the fungicides, dibutylin and triphenyltin induce adipocyte differentiation through PPARγ activation, but not the glucocorticoid receptor pathway^67^ (Table 4).

### 3.5 Conservatives

#### 3.5.1 Cellular studies

The effects of the conservative dicyclohexyl phthalate were analyzed at concentration levels of 33 and 330 μg/l on 3 T3‐L1 cells. Dicyclohexyl phthalate induced glucocorticoid receptor activity by 3.5 times at a concentration of 330 μg/l and lipid accumulation by 1.75 times at a concentration of 33 μg/l, resulting in adipocyte differentiation^49^ (Table 5).

**TABLE 5:** In vitro studies recounting the effects of conservatives.

Pollutants and concentrations	Cell type	Results	References
DCHP: 1 μmol/l and 100 nmol/l for 3 days	3 T3‐L1	DCHP stimulates GR activity DCHP stimulates adipogenesis through the GR pathway	Sargis et al, 2010
Butylparaben: 2 × 10^−4^, 10^−4^ and 5 × 10^−5^ M for 3 T3‐L1 1, 10 and 50 μM for hADSC, for 7 days	3 T3‐L1, hADSC	Parabens activate GR and do not bind competitively to GR Parabens transactivate PPARγ Parabens promote adipogenesis by activating multiple nuclear receptor including the GR and/or the PPARγ pathways Parabens modulate adipokines, leptin, and adiponectin expression Leptin expression is reduced by parabens in human adipocytes	Hu et al, 2013

Abbreviations: 3 T3‐L1, mouse embryonic fibroblasts; DCHP, Dicyclohexyl phthalate; GR, glucocorticoid receptor; hADSC, human adipose‐derived stromal cell; PPARγ, peroxisome proliferator–activated receptor.

The effects of parabens, in particular butylparaben, were investigated on 3 T3‐L1 murine adipocytes at concentration levels ranging from 10 to 19 mg/l and human adipose‐derived stromal cells (hADSC) at concentration levels ranging from 194 μg to 9.7 mg/l. A 2.3‐fold increase in 3 T3‐L1 adipocyte differentiation, a 3‐fold increase in *PPARγ* gene expression, and a 6‐fold increase in *C/EBPα* gene expression were observed at 19 mg/l. Increases in the gene expression of *leptin* (by a factor of 5.5 at 1.9 mg/l), *adiponectin* (by a factor of 2.5 at 19 mg/l), *aP2* (by a factor of 2 at 19 mg/l), and *FAS* (by a factor of 4 at 19 mg/l) were observed. A 1.5‐fold increase in glucocorticoid receptor activity was induced by butylparaben at 19 mg/l. Butylparaben also induced a 3.2‐fold increase in mRNA expression of the glucocorticoid receptor target gene *Lipin1*, which was significantly reduced by a factor of 4 in the presence of the small interfering RNA (si) siGR. Butylparaben competed for the binding of glucocorticoid receptors and did not induce adipocyte differentiation in the absence of a glucocorticoid. Additionally, glucocorticoid receptor antagonist RU486 induced decreased lipid accumulation (by a factor of 1.9), *PPARγ* (by a factor of 1.8), *C/EBPα* (by a factor of 2), and *adiponectin* (by a factor of 1.3) gene expression at 19 mg/l. As for PPARγ, the results revealed an increased gene expression of the PPARγ target *perilipin* 5 times at 19 mg/l, which was significantly reduced by 9.5 times in the presence of siPPARγ. SiPPARγ significantly reduced *aP2* gene expression by a factor 3.2. When PPARγ antagonists (GW9662, BADGE) were added to the conservatives (at 19 mg/l), 1.2‐fold and 1.4‐fold decreases in lipid accumulation were observed for each antagonist, respectively. GW9662 also decreased *PPARγ C/EBPα*, and *adiponectin* gene expression by 1.1, 1.4, and 1.1 times, respectively, while BADGE decreased *PPARγ, C/EBPα*, and *adiponectin* gene expression by 1.4, 1.9 and 1.2 times, respectively. As for hADSC, butylparaben increased lipid accumulation by 7 times and increased *adiponectin* gene expression by 22 times at 0.19 mg/l. It also increased gene expression of *aP2* by a factor of 2 at 0.19 mg/l, of 3.2 at 1.9 mg/l, and of 1.5 at 9.5 mg/l, of *FAS* and *PPARγ* by a factor of 3.5 and 3 at 0.19 mg/l, respectively, and of *C/EBPα* by a factor of 1.5 (at 0.19 and 1.9 mg/l). Conversely to what was observed in murine adipocytes, leptin gene expression was significantly reduced in human cells by 2.5 times at 9.5 mg/l. Overall, the results were magnified by adding the linear alkyl chain with an aromatic ring in benzylparaben. These results prove that parabens induce adipogenesis mediated by glucocorticoid receptor signaling^68^ (Table 5).

### 3.6 Heavy metals

#### 3.6.1 Human studies

A study was conducted on women aged between 18 and 55 years, working at brick kilns in Rawat, Pakistan, for 15 years and constantly exposed to 5.59 ± 0.03 μg/dl of nickel and 3.09 ± 0.01 μg/dl of cadmium for 8 h/day. The participants reported menstrual irregularities and a slightly reduced body mass index by a factor of 1.06. Alteration of blood parameters was also observed such as increased platelet distribution width (by a factor of 1.02), the number of reactive oxygen species (by a factor of 1.5), cholesterol level (by a factor of 1.7), triglycerides levels (by a factor of 2.4), low‐density lipoprotein levels (by a factor of 5), and plasma cortisol (by a factor of 1.4). However, peroxidase and superoxide dismutase activities decreased by 1.18 and 1.08 times, respectively. These data strongly suggest an association between heavy metal and metabolic and reproductive disorders in women mediated by hypothalamic–pituitary–adrenal‐axis dysregulation due to stressful conditions^69^ (Table 6).

**TABLE 6:** In vivo studies recounting the effects of heavy metals.

Pollutants	Concentrations	Species/ethnicity/age	Results	References
Cadmium, Nickel	Cd: 3,09 ± 0,01 μg/dl; Ni: 5,59 ± 0,03 μg/dl; exposition of 8 h/day for 15 years	Human: Pakistani women (18–55 years old)	Increased pollutant concentration is associated with metabolic and reproductive disorders in exposed women Pollutants increased blood cortisol, plasma cholesterol, LDL, and triglycerides and decreased HDL levels	David et al, 2020
Mercury, Lead, Selenium, Tin	Detection of 0.7 μg/l of Sn, 2 μg/l of Pb, 2.9 μg/l of Hg and 191.2 μg of Se	Human: Suriname pregnant women (28.2 years old, 50% African origin, 29.7% Asian origin, 20.1% mixed or other origin)	No association between heavy metals and birth outcomes No association between heavy metal and stress High stress is associated with low birthweight	Gokoel et al, 2021
Mercury	Detection of 4,7 ± 1,4 μg/g in females; 4,3 ± 1,2 μg/g in males	Animal: Polar bears (Canada, 2–30 years old)	There is a complex relationship between cortisol concentration and Hg in males Concentrations of Hg are higher in females Hair cortisol concentrations are influenced by age, Hg, and fatness in males Hair cortisol concentrations are influenced by fatness in females Hg influenced cortisol levels in males	Bechshoft et al, 2015
Mercury	Detection of 2.807 ± 0.153 μg/g dw in feather and 0.367 ± 0.013 μg/g wet weight in blood	Animal: Purple Martins (*Progne subis*, America)	Hg decreases body weight and fat mass in males Hg increases fat mass in females and old birds There is no correlation between Hg and corticosterone	Branco et al, 2022
Mercury, Selenium, Cadmium	Detection of Hg: 1,3–6,5 μg/g; Se: 9–76 μg/g; Cd: 31–309 μg/g	Animal: Eider ducks (Canada)	Concentrations of Se and Cd are higher in females No association between heavy metals and stress response found Cd is positively correlated to stress response and reduces abdominal fat mass in females Se affects the immune system, reduces corticosterone stress response, and induces body, fat, liver, and kidney mass gain in females Hg decreases body, abdominal fat, and spleen mass	Wayland et al, 2002
Mercury, Selenium, Cadmium	Hg: 1,5–9,8 μg/g; Se: 6,5–47,5 μg/g; Cd: 74–389 μg/g	Animal: Female eider ducks (Canada)	Hg decreased heart, fat, and dissection mass Cd reduced kidney, capture, and dissection mass No effect on the immune system was found No association between cortisol and Cd was found Hg affects vitamin A status by increasing retinol and retinyl palmitate	Wayland et al, 2003

Abbreviations: Cd, Cadmium; HDL, High‐density lipoprotein; Hg, Mercury; LDL, Low‐density lipoprotein; Ni, Nickel; Se, Selenium; Sn, Tin.

A prospective epidemiological cohort study was conducted from 2016 to 2019 on pregnant women living in Suriname in order to assess the effect of heavy metals and stress on birth outcomes. In this cohort, women were mostly of African ethnicity (50%), while the rest were either Asian (29.7%) or mixed/other (20.1%) and had a median body mass index of 25.9 kg/m^2^. Heavy metal analysis allowed the detection of the median concentrations of the following: 0.7 μg/l of tin, 2.9 μg/l of mercury, 191.2 μg/l of selenium, and 2.0 μg/l of lead. The results demonstrated that perceived stress negatively correlated with birthweight, individual resilience, and community commitment (r = −0.14, −0.16, and −0.16, respectively), but positively correlated with depression (r = 0.61). Birthweight also positively correlated with the Apgar score (i.e., standardized assessment for infants after delivery) and the mother's body mass index (r = 0.46 and 0.13, respectively). Heavy metals did not significantly correlate with any variables. Therefore, this study demonstrates that perceived stress and maternal body mass index strongly affect birth outcomes. The authors however failed to demonstrate any effects of the heavy metals on birth outcome^70^ (Table 6).

#### 3.6.2 Animal studies

Bechshoft et al investigated the association between mercury, cortisol, and fatness in polar bears. The hair of the female polar bears contained significantly more mercury than the males' hair (4.7 ± 1.4 μg/g and 4.3 ± 1.2 μg/g, respectively) and tended to exhibit a greater presence of cortisol (0.8 ± 0.6 pg/mg and 0.7 ± 0.5 pg/mg, respectively). Hair cortisol in males was significantly influenced by mercury, age, and fatness, as well as interactions between mercury and year, mercury and fatness, and year and fatness (all: p < 0.03). In female polar bears, only fatness was associated with mercury and cortisol (p = 0.0004), thereby suggesting a relationship between mercury and cortisol in a gender‐specific manner^71^ (Table 6).

Mercury concentration levels were assessed in adult purple martins in relation to their body condition. Measurements of corticosterone and mercury in feathers showed mean concentrations of 11.89 ± 1.794 pg/mg dry weight (dw) and 2.807 ± 0.153 μg/g dw, respectively. Both fat score and body mass (R^2^ = 0.31) were negatively correlated with mercury. Fat score was higher in females and lower in older birds, suggesting that mercury acts as an obesogen and is age‐ and sex‐dependent. However, no correlation was found between corticosterone and mercury^72^ (Table 6).

Wayland et al assessed the effect of mercury, selenium, and cadmium on the stress response, immune system, and body condition in eider ducks. The concentration levels of cadmium in the kidney were assessed from 31 to 309 μg/g, and those of mercury and selenium in the liver stood from 1.3 to 6.5 μg/g and 9 to 76 μg/g, respectively. Cadmium was correlated with an increased stressed response, measured as the rise in corticosterone concentrations following capture in females (R^2^ = 0.31) and a decreased abdominal fat mass (R^2^ = ‐0.2). As for mercury, the initial body mass, abdominal fat mass, and kidney mass were negatively correlated (R^2^ = ‐0.25, −0.5, and −0.25, respectively), while the spleen mass was positively correlated with mercury (R^2^ = 0.26). Regarding selenium, the stress response and the swelling after injection of phytohemagglutinin‐P were significantly increased in females (R^2^ = 0.31 and 0.61, respectively). Initial body mass, abdominal fat mass, kidney mass, liver mass, and spleen mass were all positively correlated with selenium (R^2^ = 0.28, 0.22, 0.03, and 0.22). The immune system status was also investigated through the heterophile:lymphocyte ratio. Heterophils are phagocytosing cells that form the first line of immune defense against bacterial pathogens in inflammatory lesions, while lymphocytes play a major role in humoral adaptive immunity and cell‐mediated adaptive immunity. Here, the ratio tended to negatively correlate with selenium (R^2^ = 0.27, p = 0.08). These data suggest that the stress induced by heavy metals may result from the alteration of the hypothalamic–pituitary–adrenal axis and the decrease in immune cells in a gender‐specific manner^73^ (Table 6).

This study was reproduced by the same authors in 2003 on female eider ducks. The concentration levels of mercury in the liver stood at 1.5 to 9.8 μg/g, and at 6.5 to 47.5 μg/g for selenium. The concentration levels of cadmium in kidneys were measured from 74 to 389 μg/g. No difference was observed between the heavy metal exposure and the duck's response to stress. Regarding the immune response, and unlike what was observed in the previous study, heavy metals did not herein correlate with the immune function significantly. Cadmium negatively correlated with initial body mass (R^2^ = ‐0.25), body mass after dissection (R^2^ = ‐0.14), and kidney mass (R^2^ = ‐0.35). As for mercury, body mass at dissection, fat mass, and heart mass was negatively correlated (R^2^ = ‐0.27, −0.33, and −0.32, respectively), although it positively correlated with retinol and retinyl palmitate (R^2^ = 0.32 and 0.37, respectively). These results suggest that heavy metals affect the body's condition and vitamin A metabolism. Unlike what was observed before, the present study failed to prove any effects of the heavy metals on the immune system and the hypothalamic–pituitary–adrenal axis^74^ (Table 6).

## 4 DISCUSSION

The main purpose of this systematic review was to investigate the complex relationship between obesity, stress, and pollution through in vivo and in vitro studies. Selective criteria were therefore defined for the studies discussing these three topics to be shortlisted. From the 1869 screened articles from PUBMED, Web of science, and EMBASE, only 42 articles truly addressed them. The number and type of studies were fully pollutant‐category dependent: the most widely studied ones were pesticides and SVOC, while conservatives and heavy metals were more rarely investigated. While studies in humans were relatively scarce, a majority of the studies were conducted in vivo on rodents and more intermittently on other wild species such as bees, polar bears, or birds. The in vitro cellular studies were mostly conducted on the same type of cell line, the 3 T3‐L1 murine preadipocyte.

We provide evidence that in most articles, pollution is responsible for stress‐response disruption and results in weight gain. Both in in vivo and in vitro studies, the majority of authors conclude that exposure to SVOC, heavy metals, conservatives, and pesticides leads to adipogenesis, weight gain, and obesity. However, few articles showed contrasted findings, illustrated by weight loss, reduced stress response due to pollutant exposure,^35^ no effect on body weight, and an absence of stress response.^36^

Semi‐volatile organic compounds, pesticides, and conservatives are generally metabolized in the liver by phase I (cytochrome P450) and phase II enzymes (UDP‐glucuronosyltransferases, sulfotransferases, N‐acetyltranseferases, and methyltransferases), then eliminated through expiration, in urine or in feces.^75^ In contrast, heavy metals metabolization requires glutathione and metallothioneins.^76^ Obesity negatively alters phase I and phase II enzymes gene expression,^77^, ^78^ while glucocorticoids positively regulate them.^79^, ^80^ Nevertheless, some pollutant traces remain in the organism and are stored in adipose tissue (e.g., organic compounds), bones, and teeth (e.g., lead), before being slowly eliminated.^81^, ^82^ Pesticides, polychlorinated biphenyls, dioxins, flame retardants, and phthalates are stored in white adipose tissue, where they interact with PPARs, the aryl hydrocarbon receptor (AhR), and steroid receptors.^83^ The PPARγ signaling pathway is a key mediator in the association between obesity and pollution.^40^, ^67^ PPARγ is a ligand‐dependent transcription factor highly expressed in adipocytes and a master regulator of preadipocyte differentiation, adipogenesis, and lipid storage.^84^ Pollutants such as phthalates^85^ and perfluorooctanoic acid^86^ activate the transcription factor PPARγ, which promotes adipocyte differentiation. Furthermore, pollutants can induce obesity through the activation of AhR.^87^ The AhR is a ligand‐activated transcription factor present in several organs. It links environmental chemical stimuli with adaptive responses, such as detoxification, cellular homeostasis, or immune response.^88^ Other pathways, such as glucocorticoids, are necessary to maintain energy availability even in the absence of stress, inducing lipolysis to release stored energy, which could account for weight variations.^89^ Conversely, the glucocorticoid receptor^90^ promotes adipogenesis through the regulation of the expression of various genes including *Klf5* and *C/EBPδ* in the presence of pollutants.^51^ Moreover, a recent study confirmed that obesity decreases glucocorticoid receptor gene expression in the adipose tissue, however, the resulting cortisol effect on adipokine secretion has not been clearly evidenced.^91^ Several pollutants (e.g., phthalates, Bisphenol A, organic pollutants) are able to cross the placental barrier, leading to fetal growth restriction, birth weight variations, thyroid and reproductive dysfunction (e.g., undescended testes, delayed puberty in males and early puberty, altered breast development in females). Additionally, endocrine‐disrupting chemicals have been associated with abnormally increased estrogen levels, leading to the up‐regulation of genes involved in adipocyte differentiation.^92^, ^93^ Pollutants (e.g., polychlorinated biphenyls, heavy metals) also affect the hypothalamic–pituitary‐thyroid‐axe by decreasing the levels of T_3_, T_4_ and TSH hormones, increasing adipokines and impairing adipocyte metabolism.^94^ The hypothalamic–pituitary–adrenal stress response triggers the release of glucocorticoids and adrenocorticotropic hormone after exposure to stress.^89^ Glucocorticoids have a pivotal role in the body's glucose, protein, and fat metabolism.^95^ The main function of the adrenocorticotropic hormone is to stimulate adrenal glands to release cortisol. Cortisol is the stress hormone that helps to maintain blood pressure, immune function, and the body's anti‐inflammatory processes.^96^ Hence, the stress response was assessed through the analysis of cortisol or corticosterone concentrations in blood,^69^ serum,^33^ hair,^71^ and feather,^72^ through behavior tests^43^ like open‐field or elevated plus maze, and analysis of the glucocorticoid receptor activity in cells.^49^ The underlying mechanisms linking pollution exposure and alteration of the stress response are poorly known. In most cases, pollution‐induced increased levels of cortisol, corticosterone, glucocorticoid receptor activity, anxiety‐like behavior, but also resulted in hypothalamic–pituitary–adrenal axis and immune‐system dysregulation. While most articles confirmed the involvement of the glucocorticoid receptor pathway in linking stress to pollution, others proved that the glucocorticoid receptor pathway was not necessary to induce a stress response^47^. Exposure to pollutants can result in a complex activation pattern of the glucocorticoid receptor signaling, explaining these controversial results. As an illustration, several pollutants such as perfluorooctane sulfonic acid and BDE increase glucocorticoid receptor activity in the presence of cortisol, while p–p’‐DDT decreases glucocorticoid receptor activity. However, when persistent organic pollutants are combined, no effect is observed on glucocorticoid receptor activity,^97^ suggesting that pollutants act differently and sometimes antagonistically when they interact with each other. Another article suggests that pollutants like the heavy metal cadmium regulate glucocorticoid receptor expression through hypothalamic–pituitary–adrenal axis activation.^98^ The discrepancies between studies may partially result from the circadian cycle of cortisol/corticosterone, which can influence the results depending on the matrix used. The cortisol concentration in hair is representative of an average stress level, whereas blood's cortisol concentration assesses an acute measurement of stress.

Regarding the link between obesity and pollution, a majority of studies in this systematic review report a positive association between pollution and weight gain.^18^, ^20^ Yet, depending on the pollutant, species, sex, ethnicity, and age, other studies reported different findings, such as weight loss,^70^ no alteration in weight or different adipose tissue fat distribution in response to pollutants.^46^ Little is known about the effect of pollutants on subcutaneous and visceral adipose tissue and no significant difference in pollutant distribution between the two types of adipose tissues has been identified. Literature tends towards a more pronounced effect of pollution on the visceral adipose tissue of females.^99^ Herein, pollutants seem to negatively alter gene expression in the visceral but not subcutaneous adipose tissue of female mice, while it positively affect gene expression in the subcutaneous adipose tissue of male mice.^37^ As for brown adipose tissue, pollutants seem to inhibit brown adipocyte tissue thermogenesis by inducing mitochondrial respiration dysfunction, favoring obesity,^66^ which is in line with the literature.^100^ Moreover, controversial results were sometimes obtained when studying the same pollutant in different species.^43^, ^46^ As an illustration, exposure to glyphosate results alternatively in obesogenic and anorexigenic effects in mice^55^ and Argentine tegu.^57^ The modifying effect of ethnicity on the association between pollutant's exposure and health outcomes is unclear. Latino^33^ and Norwegian^39^ populations with obesity had lower cortisol levels, while normal‐weight Suriname^70^ and Pakistan^69^ populations had increased cortisol levels. In contrast, multi‐ethnic (European, Latino, African American, East Asian) studies tended to associate pollutants with higher cortisol levels regardless of weight and ethnicity.^101^ Non‐monotonic effects may account for these discrepancies. As for the association between pollution, stress, and obesity, only a limited number of studies have investigated these three variables together. These studies are undeniably scarce and should be remedied in the future. Although the articles selected for this systematic review addressed obesity, pollution, and stress, the authors did not investigate the association between all three and only focused on classic associations between two variables at a time. Furthermore, among the eligible studies, the effects of low‐grade chronic versus high‐grade acute pollutant exposure on stress response and obesity have not been thoroughly investigated, therefore we cannot conclude on any potential metabolic adaptation to pollutant concentration. Elaborated statistical analyses that explore complex/triangular patterns of associations between three variables, such as mediation (e.g. Sobel test)^102^ and interaction tests^19^ have yet to be performed. This could clarify the relationships between a mediator (stress, for that matter), exposure (pollution), and outcome (obesity) and their role in these relationships.^103^

Several modifying factors such as sex, age, circadian rhythm, glucocorticoid concentrations, and pollutant measurement methods impact the association between pollution, stress, and obesity. For instance, phthalates promote metabolic syndrome preferentially in premenopausal women.^104^ Glucocorticoid concentrations are known to fluctuate diurnally, decreasing in the morning and increasing at night. Hence, the circadian rhythm exerts an important role as well and is likely to affect the concentration of circulating glucocorticoids to synchronize with the “master” circadian CLOCK.^105^ Other articles demonstrated that ambient air pollution such as particulate matter activates the hypothalamic–pituitary–adrenal axis to promote thyroid diseases, increase stress response, and induce weight loss^106^ or weight gain during pregnancy.^107^ As for measurement methods, the literature confirmed that hair is more reliable than blood or urine. It is representative of an individual's average exposure to pollutants, while concentrations in blood and urine can vary quickly over time.^108^

Our study has several strengths. This systematic review is to our knowledge the first one written with the aim of investigating the link between pollution, stress, and obesity. Only two other reviews were within close range of our study objective. In the first review, the links between obesity and stressors, including pollutants, were studied. However, the association between obesity, stress, and pollution together was not looked through, only dual associations between obesity and stress or obesity and pollutants were discussed.^109^ The second review, written about the effects of physical activity on persistent organic pollutants in obesity, briefly states that the glucocorticoid receptor, a key factor in adipogenesis, may be affected by persistent organic pollutants. However, the association between obesity, stress, and pollution as well as their underlying mechanisms, was not investigated.^110^

We completed a high‐quality, reliable, and easily readable systematic review. It is registered on PROSPERO and uses three databases for article screening with a double‐blind reading. Each paper was assessed for quality using four quality assessment tools: ToxRTools, to assess the reliability of toxicological data, BEES‐C, to evaluate the quality of research proposals and studies that incorporate biomonitoring data on short‐lived chemicals, SYRCLE‐tools, to assess methodological quality and bias that play a role in animal experiments, and the CAMARADES checklist, to assess the quality of meta‐analyses of preclinical studies. For ToxRTools and BEES‐C, the majority of the papers obtained a high‐quality score, ensuring the reliability of the data. As for the SYRCLE tools and the CAMARADES checklist, the quality assessment revealed for all papers a mean average quality scoring, which still qualifies the data as reliable with restrictions. This review includes papers conducted on humans, animals, and cells, which enables the comparison between several experimental models. It is important to note that most cellular studies used the same cellular model (3 T3‐L1 murine preadipocytes) which facilitates the comparison between the experiments. Moreover, internationally recognized experts in each field (stress, obesity, and toxicology) were involved in this systematic review.

However, several limitations should be noted in our review. The conjunction of the three topics excludes articles dealing with only two of the three topics, making the selection criteria more restrictive. There are no articles discussing the mechanistic aspects linking pollution, stress, and obesity, and mediation analyses between the three topics are yet to be done. There are countless pollutants, and only a few are currently being prioritized in research. Several pollutants were not investigated such as allyl‐2,4,6‐tribromphenylether, tetrabromoethylcyclohexane, lindane, pyrethroid, chromium, and many others. As a result, many biological pathomechanisms have yet to be discovered. Few studies have investigated the synergic or antagonistic effects of cocktails of pollutants. In this review, only five papers used a mixture of pollutants; however, these mixtures were not compared to the pollutants alone, hence we could not conclude on the effects of these cocktails. As for the study model, few studies have been carried out in humans, and they do not include all ethnic groups. Here, human studies have been carried out on populations in Europe, East Asia, and South America. It is important to note that stress response assessment was only measured once at the beginning of the study and not longitudinally throughout the experiment. This creates a bias because cortisol concentration fluctuates over time. Only selected pollutants were identified and quantified by the authors, inducing a selection bias because not all pollutants are taken into account while being present in the environment. As for obesity assessment, the body mass index measure is the main criterion used for obesity and is calculated by dividing the individual's weight by his height squared.^111^ In our systematic review, all the selected articles conducted on humans used the body mass index to assess obesity. However, recent studies point out several flaws in this measurement. Body mass index depends on sex, age, and ethnicity.^111^ Body mass index does not differentiate between bone, muscle, and fat mass, and body fat mass can vary substantially between individuals at a given body mass index.^112^ It does not inform on body fat distribution.^113^ A more reliable way to assess obesity would have been to measure the percentage of body fat mass and waist‐to‐hip ratio in addition to body mass index.^112^, ^114^ As for animal studies, many have been carried out on non‐rodents such as polar bears, birds, cats, and bees. Certain species are chosen for specific objectives, for instance, birds have been extensively documented for ecotoxicological studies due to their ability to migrate and their sensitivity to environmental pollutants.^115^ In addition, cats are often used as models for human diseases due to the wealth of information on their genome, which is homologous to humans.^116^ Regarding cellular studies, only one cell line stood out (3 T3‐L1 murine preadipocytes), while experiments on other cell lines were scarce.

**FIGURE 2:**
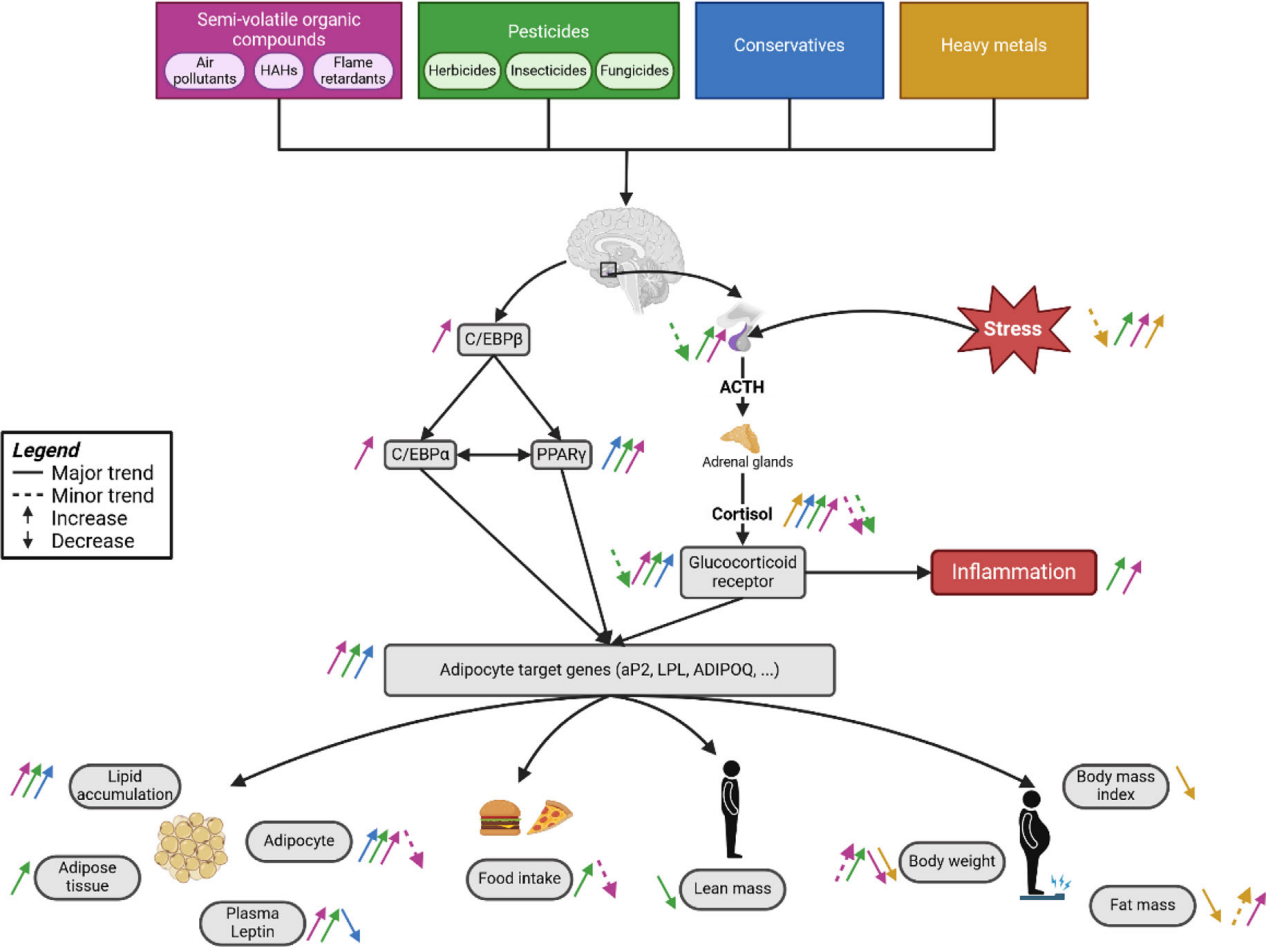
Overview of the effects of pollution on stress and obesity. The violet arrows represent the effects after exposition to semi‐volatile organic compounds, the green arrows represent the effects after exposition to pesticides, the blue arrows represent the effects after exposition to conservatives, and the yellow arrows represent the effects after exposition to heavy metals, in in vivo and in vitro studies. ACTH: adrenocorticotropic hormone; HAHs: halogenated aromatic hydrocarbons; C/EBP: CCAAT‐enhancer‐binding proteins; PPARγ: peroxisome proliferator‐activated receptor gamma. [Correction added on 30 January 2025, after first online publication: Figure 2 has been updated in this version.]

In conclusion, a growing body of evidence shows a clear association between pollution and stress, pollution and obesity, and stress and obesity in literature (Figure 2). Endocrine‐disrupting chemicals alter the glucocorticoid and PPARγ pathways to promote hyperphagia, inflammation, adipocyte hyperplasia and hypertrophy, and obesity. However, these effects depend on sex, age, and the pollutant. Future directions of research include statistical and mechanistic studies to understand the links between pollution, stress, and obesity. Enhanced methods to measure chemical exposure, biological and perceived stress, and anthropometry are in great demand. The modulation of stress response by physical activity is another original topic of investigation.^117^ In addition, little is known about the interactions between genetic predisposition and pollutant exposure on psychosocial stress and weight variations.^118^ Considering the rising exposure of global populations to pollution, psychosocial stress, and over‐nutrition, further research is warranted to reduce our environmental footprint and tackle stress and obesity at the population level.

## AUTHOR CONTRIBUTIONS

S.E.K., S.H., R‐C.D., A.vN., J.D.T, N.G., and D.M. designed the review; A.vN. and D.M. contributed to funding; S.E.K., S.H., C.M., and D.M. created the literature search; S.E.K., S.H., C.M., N.G., and D.M. conducted research; S.E.K. and D.M. conducted the quality assessment; S.E.K., S.H., N.G., and D.M. wrote the manuscript; S.E.K. designed the figure and the tables. R‐C.D., C.M., A.vN., and J.D.T. critically reviewed the manuscript for important intellectual content; N.G. and D.M. had primary responsibility for the final content and were the guarantors of the study. All authors read and approved the final manuscript.

## CONFLICT OF INTEREST STATEMENT

The authors have no conflict of interest to declare.

## Supporting information


**Table S1.** PRISMA2020 statement: Checklists.
**Table S2**. Search strategy: syntax with specific keywords.
**Table S3**. Number of participants per study.
**Table S4.** Summary of quality assessment tools score.
