# Supplementary material for: Pollution, stress response, and obesity: A systematic review
Source: Obes Rev. 2025 Jan 17;26(5):e13895. doi: 10.1111/obr.13895 (PMC11964802; doi:10.1111/obr.13895)
Supplement: Supplementary file 1 — Table S1. PRISMA2020 statement: Checklists. Table S2. Search strategy: syntax with specific keywords. Table S3. Number of participants per study. Table S4. Summary of quality assessment tools score. [file OBR-26-e13895-s001.pdf]

Title: Pollution, stress response, and obesity: a systematic review

Authors: Sandra El Kouche[1], Sarah Halvick[1,2], Chloe Morel[1], Radu-Corneliu Duca[2,3], An van Nieuwenhuyse[3,4], Jonathan D. Turner[5], Nathalie Grova[1,5,#,*], David Meyre[1,6,#,*]

Affiliations: [1]Inserm UMR 1256 Nutrition-Genetics-Environmental Risk Exposure (N-G-ERE), University of Lorraine, Nancy, France; [2]National Health Laboratory (LNS), Department of Health Protection, Unit Environmental Hygiene and Human Biological Monitoring, Dudelange, L-3555, Luxembourg; [3]KU Leuven (University of Leuven), Department of Public Health and Primary Care, Environment and Health, Leuven, 3000, Belgium; [4]National Health Laboratory (LNS), Department of Health Protection, Dudelange, L-3555, Luxembourg; [5]Immune Endocrine Epigenetics Research Group, Department of Infection and Immunity, Luxembourg Institute of Health, 29 rue Henri Koch, L-4354 Esch-sur-Alzette, Luxembourg; [6]Department of Health Research Methods, Evidence, and Impact, McMaster University, Hamilton, Ontario, Canada.

***Address for the corresponding authors:**
Pr David Meyre, Inserm UMR 1256 N-GERE (Nutrition-Genetics Environmental Risk Exposure) - University of Lorraine, Faculty of Medicine of Nancy, 54500, Vandoeuvre les Nancy, France, Tel: (33)-372746132, Email: david.meyre@univ-lorraine.fr. Dr Nathalie Grova, Inserm UMR 1256 N-GERE (Nutrition-Genetics Environmental Risk Exposure) - University of Lorraine, Faculty of Medicine of Nancy, 54500, Vandoeuvre les Nancy, France, Tel: (33)-372746141 Email: nathalie.grova@univ-lorraine.fr.

Table S1. PRIMSA2020 statement Checklists

Section and Topic	Item #	Checklist item	Location where item is reported
**TITLE**			
Title	1	Identify the report as a systematic review.	Page 1
**ABSTRACT**			
Abstract	2	See the PRISMA 2020 for Abstracts checklist.	Page 5, Table S1 (page 4)
**INTRODUCTION**			
Rationale	3	Describe the rationale for the review in the context of existing knowledge.	Page 4
Objectives	4	Provide an explicit statement of the objective(s) or question(s) the review addresses.	Page 4
**METHODS**			
Eligibility criteria	5	Specify the inclusion and exclusion criteria for the review and how studies were grouped for the syntheses.	Page 5
Information sources	6	Specify all databases, registers, websites, organisations, reference lists and other sources searched or consulted to identify studies. Specify the date when each source was last searched or consulted.	Page 5
Search strategy	7	Present the full search strategies for all databases, registers and websites, including any filters and limits used.	Page 5
Selection process	8	Specify the methods used to decide whether a study met the inclusion criteria of the review, including how many reviewers screened each record and each report retrieved, whether they worked independently, and if applicable, details of automation tools used in the process.	Page 5
Data collection process	9	Specify the methods used to collect data from reports, including how many reviewers collected data from each report, whether they worked independently, any processes for obtaining or confirming data from study investigators, and if applicable, details of automation tools used in the process.	Page 5
Data items	10a	List and define all outcomes for which data were sought. Specify whether all results that were compatible with each outcome domain in each study were sought (e.g. for all measures, time points, analyses), and if not, the methods used to decide which results to collect.	Page 5
	10b	List and define all other variables for which data were sought (e.g. participant and intervention characteristics, funding sources). Describe any assumptions made about any missing or unclear information.	Page 5
Study risk of bias assessment	11	Specify the methods used to assess risk of bias in the included studies, including details of the tool(s) used, how many reviewers assessed each study and whether they worked independently, and if applicable, details of automation tools used in the process.	Page 6
Effect measures	12	Specify for each outcome the effect measure(s) (e.g. risk ratio, mean difference) used in the synthesis or presentation of results.	Page 5
Synthesis methods	13a	Describe the processes used to decide which studies were eligible for each synthesis (e.g. tabulating the study intervention characteristics and comparing against the planned groups for each synthesis (item #5)).	Page 5
	13b	Describe any methods required to prepare the data for presentation or synthesis, such as handling of missing summary statistics, or data conversions.	Page 5
	13c	Describe any methods used to tabulate or visually display results of individual studies and syntheses.	Page 5
	13d	Describe any methods used to synthesize results and provide a rationale for the choice(s). If meta-analysis was performed, describe the model(s), method(s) to identify the presence and extent of statistical heterogeneity, and software package(s) used.	Page 6

Section and Topic	Item #	Checklist item	Location where item is reported
	13e	Describe any methods used to explore possible causes of heterogeneity among study results (e.g. subgroup analysis, meta-regression).	Page 5
	13f	Describe any sensitivity analyses conducted to assess robustness of the synthesized results.	Page 6
Reporting bias assessment	14	Describe any methods used to assess risk of bias due to missing results in a synthesis (arising from reporting biases).	Page 6
Certainty assessment	15	Describe any methods used to assess certainty (or confidence) in the body of evidence for an outcome.	Page 6
**RESULTS**			
Study selection	16a	Describe the results of the search and selection process, from the number of records identified in the search to the number of studies included in the review, ideally using a flow diagram.	Page 7, figure 1
	16b	Cite studies that might appear to meet the inclusion criteria, but which were excluded, and explain why they were excluded.	Page 7, figure 1
Study characteristics	17	Cite each included study and present its characteristics.	Tables 1 (page 16-18), 2 (pages 19-20), 3 (pages 28-30), 4 (pages 30-31), 5 (page 3) and 6 (pages 36-37)
Risk of bias in studies	18	Present assessments of risk of bias for each included study.	Page 8
Results of individual studies	19	For all outcomes, present, for each study: (a) summary statistics for each group (where appropriate) and (b) an effect estimate and its precision (e.g. confidence/credible interval), ideally using structured tables or plots.	Pages 8-37
Results of syntheses	20a	For each synthesis, briefly summarise the characteristics and risk of bias among contributing studies.	Pages 8-37
	20b	Present results of all statistical syntheses conducted. If meta-analysis was done, present for each the summary estimate and its precision (e.g. confidence/credible interval) and measures of statistical heterogeneity. If comparing groups, describe the direction of the effect.	Pages 8-37
	20c	Present results of all investigations of possible causes of heterogeneity among study results.	Pages 8-37
	20d	Present results of all sensitivity analyses conducted to assess the robustness of the synthesized results.	Pages 8-37
Reporting biases	21	Present assessments of risk of bias due to missing results (arising from reporting biases) for each synthesis assessed.	Pages 8-37
Certainty of evidence	22	Present assessments of certainty (or confidence) in the body of evidence for each outcome assessed.	Pages 8-37
**DISCUSSION**			
Discussion	23a	Provide a general interpretation of the results in the context of other evidence.	Page 38
	23b	Discuss any limitations of the evidence included in the review.	Pages 40-41
	23c	Discuss any limitations of the review processes used.	Pages 40-41
	23d	Discuss implications of the results for practice, policy, and future research.	Page 41
**OTHER INFORMATION**			

Section and Topic	Item #	Checklist item	Location where item is reported
Registration and protocol	24a	Provide registration information for the review, including register name and registration number, or state that the review was not registered.	Pages 2 and 5
	24b	Indicate where the review protocol can be accessed, or state that a protocol was not prepared.	Page 5
	24c	Describe and explain any amendments to information provided at registration or in the protocol.	Page 5
Support	25	Describe sources of financial or non-financial support for the review, and the role of the funders or sponsors in the review.	Page 43
Competing interests	26	Declare any competing interests of review authors.	Page 43
Availability of data, code and other materials	27	Report which of the following are publicly available and where they can be found: template data collection forms; data extracted from included studies; data used for all analyses; analytic code; any other materials used in the review.	Page 43

From: Page MJ, McKenzie JE, Bossuyt PM, Boutron I, Hoffmann TC, Mulrow CD, et al. The PRISMA 2020 statement: an updated guideline for reporting systematic reviews. BMJ 2021;372:n71.doi: 10.1136/bmj.n71
For more information, visit:http://www.prisma-statement.org/

PRISMA 2020 for Abstracts Checklist

Section and Topic	Item #	Checklist item	Reported (Yes/No)
**TITLE**			
Title	1	Identify the report as a systematic review.	Yes
**BACKGROUND**			
Objectives	2	Provide an explicit statement of the main objective(s) or question(s) the review addresses.	Yes
**METHODS**			
Eligibility criteria	3	Specify the inclusion and exclusion criteria for the review.	Yes
Information sources	4	Specify the information sources (e.g. databases, registers) used to identify studies and the date when each was last searched.	Yes
Risk of bias	5	Specify the methods used to assess risk of bias in the included studies.	Yes
Synthesis of results	6	Specify the methods used to present and synthesise results.	Yes
**RESULTS**			
Included studies	7	Give the total number of included studies and participants and summarise relevant characteristics of studies.	Yes
Synthesis of results	8	Present results for main outcomes, preferably indicating the number of included studies and participants for each. If meta-analysis was done, report the summary estimate and confidence/credible interval. If comparing groups, indicate the direction of the effect (i.e. which group is favoured).	Yes
**DISCUSSION**			
Limitations of evidence	9	Provide a brief summary of the limitations of the evidence included in the review (e.g. study risk of bias, inconsistency and imprecision).	Yes
Interpretation	10	Provide a general interpretation of the results and important implications.	Yes
**OTHER**			
Funding	11	Specify the primary source of funding for the review.	N/A
Registration	12	Provide the register name and registration number.	Yes

From: Page MJ, McKenzie JE, Bossuyt PM, Boutron I, Hoffmann TC, Mulrow CD, et al. The PRISMA 2020 statement: an updated guideline for reporting systematic reviews. BMJ 2021;372:n71. doi: 10.1136/bmj.n71

For more information, visit: http://www.prisma-statement.org/

Table S2. Search strategy syntax with specific keywords

Database	Keywords
PUBMED	((obesity OR adipose tissue OR BMI OR body mass index OR fat OR obese OR overweight) AND (stress OR corticoid OR corticosteroid OR corticosterone OR cortisol OR cortisone OR glucocorticoid OR glucocorticosteroid OR hypothalamic–pituitary–adrenal) AND (pollutant OR persistent organic pollutant OR endocrine disruptor OR indoor pollutant OR sVOC OR semivolatile organic compound OR flame retardant OR fire retardant OR polybrominated diphenyl ether OR polychlorobiphenyl OR perfluoroalkyl acid OR polyfluorinated alkyl OR chlorinated paraffin OR polycyclic aromatic hydrocarbon OR biocide OR pesticide OR insecticide OR heavy metal) NOT (archaea OR bacteria OR cellular stress OR endoplasmic reticulum OR er stress OR fungus OR oxidative stress OR plant OR post-traumatic stress OR pulmonary arterial hypertension OR reticulum stress))
Web Of Science	(ALL=(obesity) OR ALL=(adipose tissue) OR ALL=(BMI) OR ALL=(body mass index) OR ALL=(fat) OR ALL=(obese) OR ALL=(overweight)) AND (ALL=(stress) OR ALL=(corticoid) OR ALL=(corticosteroid) OR ALL=(corticosterone) OR ALL=(cortisol) OR ALL=(cortisone) OR ALL=(glucocorticoid) OR ALL=(glucocorticosteroid) OR ALL=(hypothalamic–pituitary–adrenal)) AND (ALL=(pollutant) OR ALL=(persistent organic pollutant) OR ALL=(endocrine disruptor) OR ALL=(indoor pollutant) OR ALL=(sVOC) OR ALL=(semivolatile organic compound) OR ALL=(flame retardant) OR ALL=(fire retardant) OR ALL=(polybrominated diphenyl ether) OR ALL=(polychlorobiphenyl) OR ALL=(perfluoroalkyl acid) OR ALL=(polyfluorinated alkyl) OR ALL=(chlorinated paraffin) OR ALL=(polycyclic aromatic hydrocarbon) OR ALL=(biocide) OR ALL=(pesticide) OR ALL=(insecticide) OR ALL=(heavy metal)) NOT (ALL=(archaea) OR ALL=(bacteria) OR ALL=(cellular stress) OR ALL=(endoplasmic reticulum) OR ALL=(er stress) OR ALL=(fungus) OR ALL=(oxidative stress) OR ALL=(plant) OR ALL=(post-traumatic stress) OR ALL=(pulmonary arterial hypertension) OR ALL=(reticulum stress))
EMBASE	('obesity' OR 'adipose tissue' OR 'bmi' OR 'body mass index' OR 'fat' OR 'obese' OR 'overweight') AND ('stress' OR 'corticoid' OR 'corticosteroid' OR 'corticosterone' OR 'cortisol' OR 'cortisone' OR 'glucocorticoid' OR 'glucocorticosteroid' OR 'hypothalamic–pituitary–adrenal') AND ('pollutant' OR 'persistent organic pollutant' OR 'endocrine disruptor' OR 'indoor pollutant' OR 'svoc' OR 'semivolatile organic compound' OR 'flame retardant' OR 'fire retardant' OR 'polybrominated diphenyl ether' OR 'polychlorobiphenyl' OR 'perfluoroalkyl acid' OR 'polyfluorinated alkyl' OR 'chlorinated paraffin' OR 'polycyclic aromatic hydrocarbon' OR 'biocide' OR 'pesticide' OR 'insecticide' OR 'heavy metal') NOT ('archaea' OR 'bacteria' OR 'cellular stress' OR 'endoplasmic reticulum' OR 'er stress' OR 'fungus' OR 'oxidative stress' OR 'plant' OR 'post-traumatic stress' OR 'pulmonary arterial hypertension' OR 'reticulum stress')

Table S3. Number of participants per study

Publication Year	Author	Model(s)		Number of animals	Number of Humans
2015	Bechshoft, T.	animal		378	
2022	Branco, Jonathan M.	animal		79	
2012	Chang, Ling-Ling	animal		60	
2018	Chen, Yana	animal	cellular	43	
2023	Dangudubiyyam, Sri Vidya	animal		24	
2023	Elizabeth Deeter, Megan	animal		1806	
2021	Huang, Wei-Chi	animal		12	
1987	Jovanovich, Lela	animal		55	
2023	Khidkhan, Kraisiri	animal		14	
2015	Kim, Benjamin	animal		20	
2023	Lama, A.	animal		48	
2015	Lyssimachou, Angeliki	animal		32	
2011	Maia, Lucas O.	animal		32	
2016	Merlo, Eduardo	animal		66	
2021	Merrill, Alyssa K.	animal		55	
2019	Mestre, Ana P.	animal		72	
2015	Regnier, Shane M.	animal		56	
2024	Rosolen, Ana Paula Farina	animal		91	
2021	Vega, Nathalie	animal		15	
2002	Wayland, M.	animal		39	
2003	Wayland, Mark	animal		21	
2016	Yang, Minglan	animal	human	21	228
2023	Yavuz, Yavuz	animal		23	
2011	Zaya, Renee M.	animal		61	
2013	Zimmer, Karin E.	animal		40	
2014	Atlas, Ella	cellular			
2019	Biserni, Martina	cellular			
2014	Boucher, J G	cellular			
2018	Chappell, Vesna A	cellular			
2013	Hu, Pan	cellular			
2020	Liu, Qian S.	cellular			
2013	Neel, Brian A.	cellular			
1995	Phillips, Marjorie	cellular			
2010	Sargis, Robert M.	cellular			
2023	Ticiani, Elvis	cellular			
2022	Völker, Johannes	cellular			
2013	Wang, J	cellular			
2020	David, Mehwish	human			232

2021	Gokoel	human			384
2020	Jansen, Aina	human			34
2022	Li, Lei	human			7419
2021	Toledo-Corral, C. M.	human			203
Total				3163	8500

Table S4. Summary of quality assessment tools score

	Quality assessment tool	ToxRTool (out of 21 in vivo, 18 in vitro)	CAMARADES Checklists (out of 10)	SYRCLE -tools (out of 10)	BEES-C instrument score (out of 3 - lower better)
Species	Authors, year				
Human	Yang et al., 2016	20	N/A	N/A	Tier 1
	David et al., 2020	21	N/A	N/A	Tier 1
	Gokoel et al., 2021	21	N/A	N/A	Tier 1
	Jansen et al., 2020	21	N/A	N/A	Tier 1
	Li et al., 2022	21	N/A	N/A	Tier 1
	Toledo-Corral et al., 2021	21	N/A	N/A	Tier 2
Animal	Bechshoft et al., 2015	21	6	10	N/A
	Branco et al., 2022	21	7	10	N/A
	Chang et al., 2012	20	5	4	N/A
	Chen et al., 2018	18	4	4	N/A
	Dangudubiyyam et al., 2023	20	7	4	N/A
	Deeter et al., 2023	20	6	4	N/A
	Huang et al., 2021	19	6	4	N/A
	Jovanovich et al., 1987	21	2	4	N/A
	Khidkhan et al., 2023	21	5	4	N/A
	Kim et al., 2015	21	5	4	N/A
	Lama et al., 2023	21	5	3	N/A
	Lyssimachou et al., 2015	21	7	4	N/A
	Maia et al., 2011	19	5	4	N/A
	Merlo et al., 2016	21	4	3	N/A
	Merrill et al., 2021	21	4	4	N/A
	Mestre et al., 2019	19	5	4	N/A
	Regnier et al., 2015	20	5	3	N/A
	Rosolen et al., 2024	20	5	3	N/A

	Vega et al., 2021	20	6	5	N/A
	Wayland et al., 2002	20	4	9	N/A
	Wayland et al., 2003	20	3	9	N/A
	Yang et al., 2016	20	6	4	N/A
	Yavuz et al., 2023	20	6	4	N/A
	Zaya et al., 2011	19	5	4	N/A
	Zimmer et al., 2013	21	5	4	N/A
Cell	Chen et al., 2018	14	N/A	N/A	N/A
	Atlas et al., 2014	14	N/A	N/A	N/A
	Biserni et al., 2019	17	N/A	N/A	N/A
	Boucher et al., 2014	17	N/A	N/A	N/A
	Chappell et al., 2018	16	N/A	N/A	N/A
	Hu et al., 2013	15	N/A	N/A	N/A
	Liu et al., 2020	17	N/A	N/A	N/A
	Neel et al., 2013	16	N/A	N/A	N/A
	Phillips et al., 1995	13	N/A	N/A	N/A
	Sargis et al., 2010	17	N/A	N/A	N/A
	Ticiani et al., 2023	18	N/A	N/A	N/A
	Völker et al., 2022	16	N/A	N/A	N/A
	Wang et al., 2013	16	N/A	N/A	N/A
	Mean	19	5	5	Tier 1
